# The molecular architecture distinctions between compression, opposite and normal wood of *Pinus radiata*

**DOI:** 10.3389/fpls.2025.1576928

**Published:** 2025-05-13

**Authors:** Rosalie Cresswell, Alan Dickson, Michael Robertson, Suzanne Gallagher, Regis Risani, Marie Joo Le Guen, Henry Temple, Aleksandra Liszka, Lloyd Donaldson, Nigel Kirby, John Ralph, Stefan Hill, Paul Dupree, Ray Dupree, Mathias Sorieul

**Affiliations:** ^1^ Physics Department, University of Warwick, Coventry, United Kingdom; ^2^ Forest Genetics and Biotechnology, Scion, Rotorua, New Zealand; ^3^ Sainsbury Laboratory, University of Cambridge, Cambridge, United Kingdom; ^4^ Department of Plant Biotechnology, Faculty of Biochemistry, Biophysics and Biotechnology, Jagiellonian University, Krakow, Poland; ^5^ Doctoral School of Exact and Natural Sciences, Jagiellonian University, Krakow, Poland; ^6^ SAXS/WAXS Beamline, Australian Synchrotron, Australian Nuclear Science and Technology Organisation, Melbourne, VIC, Australia; ^7^ The Department of Biochemistry, The Wisconsin Energy Institute and the United States Department of Energy’s (DOE) Great Lakes Bioenergy Research Center, University of Wisconsin-Madison, Madison, WI, United States; ^8^ Department of Biochemistry, University of Cambridge, Cambridge, United Kingdom

**Keywords:** solid state NMR (ssNMR), compression wood (CW), secondary cell wall (SCW), opposite wood (OW), *Pinus radiata* (Monterey pine)

## Abstract

In gymnosperms compression wood is a specialised type of structural cell wall formed in response to biomechanical stresses. The differences in terms of gross structure, ultrastructure and chemistry are well-known. However, the differences between compression wood, normal wood, and opposite wood regarding the arrangements and interactions of the various polymers and water within their cell walls still needs to be established. The analysis of ^13^C-labelled *Pinus radiata* by solid-state NMR spectroscopy and other complementary techniques revealed several new aspects of compression and opposite wood molecular architecture. Compared to normal wood, compression wood has a lower water content, its overall nanoporosity is reduced, and the water and matrix polymers have a lower molecular mobility. Galactan, which is a specific marker of compression wood, is broadly distributed within the cell wall, disordered, and not aligned with cellulose, and is found to be in close proximity to xylan. Dehydroabietic acid (a resin acid) is immobilised and close to the H-lignin only in compression wood. Although the overall molecular mobility of normal wood and opposite wood are similar, opposite wood has different arabinose conformations, a large increase in the amount of chain ends, contains significantly more galactan and has additional unassigned mobile components highlighting the different molecular arrangement of cell wall polymers in opposite and normal wood.

## Introduction

Wood formation is a dynamic, continuous, and highly regulated process resulting in different cell and tissue types. The resulting heterogeneous structure enables trees to functionally adapt to environmental changes. Reaction wood, a specialized wood tissue, is produced in response to gravitational and mechanical stimulation and/or a change in illumination. In coniferous gymnosperms, in which the bulk of the biomass is composed of water-conducting tracheids, the lower side of the stem or branches subjected to bending stresses form a reaction wood named compression wood (Timell, 1986; Donaldson et al., 2004; Salem et al., 2023). The upper side of the stem, designated as opposite wood, has been thought to be chemically very similar to normal wood (Timell, 1986). The production of compression wood is genetically controlled as its formation inhibits the development of spiral grain and resin canals (Thomas et al., 2022). In terms of anatomical structure and compositional chemistry the transition from normal to mild to severe compression wood is a continuum (Donaldson et al., 1999; Plomion et al., 2000; Yamashita et al., 2007).

Severe compression wood can be easily identified thanks to a reddish colouration. **Supplementary Table S1** provides an overview of the structural and supramolecular differences between normal and compression wood. Compression wood tracheids are shorter, they have smaller diameters, thicker cell walls (smaller lumen) and their rounder cross-section leads to the creation of enlarged intercellular spaces. These changes in tracheid morphology create a wood with higher wall area fraction, leading to compression wood having a 15-27% increase in density (Timell, 1986; Klement et al., 2019; Sinn et al., 2022) compared to normal wood. However, at similar wall density the mechanical strength of compression wood is significantly lower than that of normal wood (Peng et al., 2019). The layout of the compression wood secondary cell wall is also very different with the disappearance of the S3 layer and formation of helical cavities in the S2 layer. At the supramolecular level, the differences in cellulose structure can be seen in the microfibril angle (MFA), microfibril diameter, and crystallite width. In normal mature wood the S2 layer has a 5-30° MFA, whereas it is 30-45° in compression wood (Donaldson et al., 2004). The dehydrated macrofibrils are reported to be larger in compression wood (22 nm) compared to normal wood (19 nm) (Donaldson, 2007). It is also reported that the cross-sectional dimension of crystalline domains is slightly smaller for compression than for opposite wood (Marton, 1972; Tanaka et al., 1981). Electron tomography reveals more kinks along the microfibrils, creating weak points due to dislocations (Xu et al., 2011). In contrast to compression wood, opposite wood has long and angular tracheids with a thick S3 layer containing helical thickenings (Timell, 1986).

The chemical compositional changes in compression wood, compared to normal wood are reported in **Supplementary Table S2**. The main changes are of three types: 1) a large increase of lignin concomitant with 2) a reduction of cellulose and 3) the appearance of new polysaccharides. In coniferous normal wood lignin almost exclusively originates from monolignol coniferyl alcohol (CA) producing a guaiacyl (G)-type lignin. The highly lignified S2 layer (S2L) of compression wood is specifically enriched in *p*-hydroxyphenyl H-type units derived from *p*-coumaryl alcohol (PA) (Fukushima and Terashima, 1991; Donaldson, 2001; Tokareva et al., 2007; Donaldson and Radotic, 2013; Zhang et al., 2017). The radical polymerisation of *p*-hydroxyphenyl units, which lack aromatic methoxy substituents, enables formation of more complex and stiffer lignin structures (Önnerud and Gellerstedt, 2003). This is consistent with a greater proportion of condensed structures found in compression wood lignin (Sakakibara, 1980; Kutsuki and Higuchi, 1981; Fagerstedt et al., 2015; Peng et al., 2019). The G-type and H-type lignin deposition patterns shift from the compound middle lamella (CML) and S3 in normal wood to exclusively S2L in compression wood and are associated with specific laccase spatial localisation and activities (Hiraide et al., 2021).

Another striking feature is the appearance of a relatively high proportion of galactan chains with up to 10% found in severe compression wood (Timell, 1986). The galactan backbone comprises 200 to 380 β-1,4-linked galactosyl moieties in pyranosidic conformation (Timell, 1986; Nanayakkara, 2007). This galactan is largely unbranched (Laine et al., 2004; Nanayakkara, 2007) but in some cases has been reported to be decorated at the C-6 position with a single β-d-galacturonic acid residue or a d-glucuronic acid (Timell, 1964; Jiang and Timell, 1972). In compression wood the spatial distribution of galactan epitopes co-localise with the lignin found in S2L (Altaner et al., 2007; Möller and Singh, 2007; Donaldson and Knox, 2012; Kim et al., 2022). As the formation and location of lignin and (1→4)-β-galactan are closely related in the S2L (Zhang et al., 2018), it has been suggested that lignin and (1→4)-β-galactan are covalently linked (Watanabe et al., 1989; Chavan et al., 2015). Another hypothesis often accepted, is that compression wood galactan could be side-chains of the pectic polysaccharide rhamnogalacturonan I (RG I) (Altaner et al., 2010; Zhang et al., 2016; Donev et al., 2018). In spruce, RG-I is linked to arabino(4-*O*-methylglucurono)xylan (AGX) via a Xyl-(1→4)-Rha linkage (Makarova and Shakhmatov, 2024), and two RG-I molecules can be covalently crosslinked to one arabinogalactan protein (AGP) in Arabidopsis (Tan et al., 2023). The high water binding capacity of galactan generates a gel-like structure modulating the cell wall matrix architecture and functioning to modulate cell elongation, water retention, and the creation of stress bearing structures (Pérez García et al., 2012; Ciancia et al., 2020; Prabhakar et al., 2023). Modulating of the length and conformation of the RG-I’s galactan side chains (e.g. in the “tree in lawn model”) influences their hydration capacity and ability to form strong hydrogels with elastomeric and hyper-elastic properties (Klaassen and Trindade, 2020; Gorshkova et al., 2022; Feng et al., 2023).

Compression wood hemicelluloses are less abundant but their distribution is similar to normal wood (Yeh et al., 2006). The *O*-acetyl-galactoglucomannans (acGGM) are mainly present in the S1 and S2 layers (Donaldson and Knox, 2012). Although acGGM content is significantly reduced (Nanayakkara et al., 2009) in severe compression wood, the AGX are only slightly reduced (Yeh et al., 2006). The AGXs are detected throughout the secondary cell wall with the more decorated molecules present in the S1 layer (Altaner et al., 2010). Laricinan/callose a (1→3)-β-glucan) is also specifically detected in the helical cavities of the S2i (S2 interior) and α(1–5)-arabinan is associated with intercellular spaces (Altaner et al., 2007).

One-dimensional solid-state NMR (ss-NMR) of compression wood (Newman, 2004) showed an increase in lignin and galactan with only minor differences in the cellulose spectrum compared to normal wood. Polarised FT-IR has previously suggested that, in coniferous secondary cell walls (SCW), acGGM has a parallel orientation with respect to the cellulose microfibrils (Peng et al., 2019) and acGGM is reported to have a greater degree of alignment than AGX. The orientation of lignin along cellulose microfibrils is increased in compression wood (Peng et al., 2019).

In terms of mechanical properties whereas compression wood has a low tensile strength and high compressive strength, opposite wood has the reverse characteristics of high tensile strength and low compressive strength. Normal wood has intermediate properties (Timell, 1986). Wood shrinks during drying (Glass and Zelinka, 2021). Compared to normal wood, the shrinkage in compression wood is much higher in all directions (+32% radial, +37% tangential and +300% longitudinal) (Zhang et al., 2018). Although the mechanical function of (1→4)-β-galactan in compression wood is currently not understood, its presence is positively associated with longitudinal shrinkage (Brennan et al., 2012; Zhang et al., 2018). The high hygroscopicity of (1→4)-β-galactan, coupled with the high MFA (possibly due a greater deposition of hemicelluloses and lignin along the microfibrils) and stiffer lignin (due to higher condensation) are possible reasons for the increased swelling in the longitudinal direction, increased brittleness, reduced tensile strength as well as the reduced modulus of elasticity of compression wood (Ryden et al., 2000; Plomion et al., 2000; Önnerud and Gellerstedt, 2003; Turnbull et al., 2005; Harris and Smith, 2006; Zhan et al., 2021).

Although compression wood offers advantages such as better shear strength, lower water uptake, and increased durability against fungi, its drawbacks limit its industrial applications (Wimmer and Johansson, 2013). The higher lignin content necessitates more chemical input and reduces pulp yield (Peng et al., 2019). Its increased hardness and brittleness pose challenges in sawmills, and its low stiffness and tendency for catastrophic failure make it unsuitable for structural uses (Sorensson and Lausberg, 1996; Timell, 1986). Additionally, the heterogeneous presence of compression and normal wood can cause distortion during drying or in service due to differential shrinkage and swelling (Sharma and Altaner, 2014) (Leonardon et al., 2010).

A deeper understanding of the structural interactions between the cell wall polymers in compression wood is therefore essential to:

- define molecular targets for softwood marker-assisted genetic improvement of timber,
- create synthetic biology strategies for improving softwood biomass’ biorefinery performance,
- design improved enzymatic biorefining processes that can effectively process compression wood.

This study aims to better understand the native molecular architecture of compression wood. Amongst the approaches used, fully ^13^C-labelled compression wood was produced for the first time and analysed, primarily by solid state NMR (ss-NMR). Having the secondary cell wall highly enriched in ^13^C (>97%) provides an increase in carbon-carbon correlation sensitivity of two orders of magnitude compared with natural abundance wood. This sensitivity gain allows the use of multidimensional ss-NMR to gain further understanding of the softwood polymer structures and, importantly, the interactions between the polymers that dictate their functionality in secondary cell walls (Kirui et al., 2022). Recent 2D solid-state NMR studies have resolved the molecular architecture of the cell wall in normal softwood (Terrett et al., 2019; Cresswell et al., 2021). The novelty of our investigation comes from our comparison between compression wood, opposite wood and normal wood in terms of:

- The difference in molecular rigidity, mobility, and order of the polymers,
- The proximity of these polymers to water,
- The relative proximity between these polymers,
- The difference in nano-porosity of the wood types.

## Materials and methods

### Production and ^13^C-labeling of *P. radiata* cuttings for NMR

Small stem cuttings (∼5 cm) were produced by tree nursery Kools Sierteeltkwekerij (Deurne, The Netherlands) from 2-year-old *P. radiata* trees. The cuttings were placed in small pots (9 × 9 cm) filled with a mixture of 1/3 vermiculite, 1/3 perlite, and 1/3 rockwool. After 7 months, callus tissue had developed, and the cuttings were then transferred to the labelling facility of IsoLife (Wageningen, The Netherlands). The cuttings were either grown straight or bent horizontally using metal wire and left to grow for 6 months in an atmosphere containing ^13^CO_2_ (99 atom% ^13^C). Straight grown trees were used for collection of normal wood samples. Compression wood and opposite wood were collected from bent trees.

### Sample collection and storage of ^13^C- labelled wood material for solid-state NMR

After taking the *P. radiata* plantlets (10–15 cm) out of the labelling facility, the stems were cut, debarked, compression wood and opposite wood separated with a blade, then snap-frozen in liquid nitrogen. The samples were then kept frozen until analysis. This wood material is considered as never dried.

### Production of *P. radiata* compression wood for non NMR analysis (nanoporosity, X-Ray diffraction, GC-MS)

Clonal *Pinus radiata* plantlets (~15–20 cm) were grown in a glasshouse, straight or bent horizontally using metal wire, and left to grow for 6 months prior analysis. For both conditions three plants (~40 cm) were collected. Normal wood was collected at the base of the straight plants (1–2 cm above soil). The compression wood was selected where the curvature was the strongest. Sections of wood (1 cm) were cut from the stem using secateurs and debarked.

### Sample Preparation for solution-state NMR

Frozen stems from the samples were cut into small pieces (10 × 2 mm), lyophilized, and 400 mg was pre-ground for 1.5 min in corrosion-resistant stainless-steel screw-top grinding jars (50 mL) containing a single steel ball bearing (30 mm) using a Retsch MM400 mixer mill. Cell wall material was isolated in 50 mL centrifuge tubes using solvents, following a method similar to the alcohol-insoluble residue (AIR) approach. The samples were sonicated for 20 min three times with each of the following solvents: distilled water, 80% ethanol, and acetone. The solvents were removed by centrifuging for 20 min and decanting. The solvent-extracted material was then freeze-dried. Ball-milling of the dry pre-extracted samples (300 or 350 mg) was via a Fritsch Pulverisette 7 Premium Line with 50 mL ZrO_2_ grinding jars and 10 × 10 mm ZrO_2_ ball-bearings at 600 rpm for a total 1 h 15 min (5 min grinding time, 5 min break, 8 cycles). The balled-milled cell wall material (50–60 mg) was collected from the jar and transferred as a dry powder directly into a 5 mm NMR tube for solution state NMR. Pre-mixed DMSO-d_6_/pyridine-d_5_ (4:1 v/v, 500 μL) was directly added into the NMR tubes. A cylindrical magnet was used inside the tube to mix the sample until the gel became homogeneous. The final sample height in the tube was ~4–5 cm. The magnet was removed from the NMR tube before running spectra on a 700 MHz NMR spectrometer described below in the solution state NMR section. As these spectra were acquired only to verify the expected lignin and polysaccharide signature differences between the three samples, and because material was limited and to be used elsewhere for enzyme lignin preparation, no replication was carried out.

### Solid-state NMR

All softwood samples were cut into slivers with a razor and packed into a 3.2 mm Magic Angle Spinning (MAS) NMR rotor. Solid-state NMR experiments were performed using either an Avance Neo or an Avance II+ (Bruker, Karlsruhe, Germany) spectrometer both operating at ^1^H and ^13^C Larmor frequencies of 600.1 and 150.9 MHz, respectively. Experiments were conducted at room temperature at MAS frequencies between 10 and 12.5 kHz. The ^13^C chemical shift was determined using the carbonyl peak at 177.8 ppm of l-alanine as an external reference with respect to tetramethylsilane (TMS); 90° pulse lengths were typically 3.0–3.5 µs (^1^H) and 3.5–5.0 µs (^13^C). Both ^1^H–^13^C cross polarisation (CP) with ramped (70–100%) ^1^H rf amplitude and a contact time of 1 ms and direct polarisation (DP) were used to obtain the initial transverse magnetization (Metz et al., 1994). The CP experiments emphasize the more rigid material, while a short, 2 s, recycle delay DP experiment was used to preferentially detect the more mobile components and a 20 s delay was used for more quantitative experiments chosen based on the ^13^C *T*_1_ of never-dried pine in our previous work (Cresswell et al., 2021). SPINAL-64 decoupling was applied during acquisition at a ^1^H nutation frequency of 70–82 kHz (Fung et al., 2000). Water-edited CP experiments (Luo and Hong, 2010) had a total proton filter time of 2 ms whilst the diffusion delay was varied from 1 to 49 ms. The non-quaternary suppression (NQS) experiment (Opella and Frey, 1979) had a total dephasing delay of 92 µs to minimise the polysaccharide signal. Intermolecular contacts were probed using 2D ^13^C–^13^C PDSD experiments with mixing times of 30 ms and 400 ms (Takegoshi et al., 2001). The acquisition time in the indirect dimension (*t*_1_) of the CP-PDSD experiments was 6.7–7.8 ms. The sweep width in the indirect dimension was between 40 and 50 kHz with 64 acquisitions per *t*_1_ and a recycle delay of 2 s. Two-dimensional double quantum (DQ) correlation spectra were recorded using either a DP or a CP refocused INADEQUATE pulse sequence which correlates directly covalently bonded carbon nuclei (Lesage et al., 1997). The acquisition time in *t*_1_ was between 5 and 7.2 ms and the carbon 90° and 180° pulse lengths were 4 and 8 µs, respectively. The 2τ spin-echo evolution time was 4.66 ms for a (π-τ-π)/2 spin echo with SPINAL-64 ^1^H decoupling applied during both the evolution and signal acquisition periods. The sweep width in the indirect dimension was 48 kHz with 128–160 acquisitions per *t*_1_ and recycle delay of 2 s. The 2D spectra were obtained by Fourier transformation into 4k (*F*_2_) × 2k (*F*_1_) points with an exponential line broadening of 50 Hz for CP and 20 Hz for DP in *F*_2_ and squared sine bell processing in *F*_1_. All spectra obtained were processed and analysed using Bruker Topspin version 3.6.

### Solution-state NMR

The solution-state NMR experiments were performed and the data were analyzed as previously reported (Kim and Ralph, 2010; Mansfield et al., 2012). The NMR spectra of the whole cell materials were acquired on a Bruker Biospin (Billerica, MA) Avance 700 MHz spectrometer equipped with a 5-mm QCI ^1^H/^31^P/^13^C/^15^N cryoprobe with inverse geometry (proton coils closest to the sample). DMSO-*d_6_*:pyridine-*d_5_* (4:1, v/v) was the solvent, and the central DMSO solvent peak (δ_C_ 39.5, δ_H_ 2.49 ppm) was used as the internal reference. An adiabatic ^1^H–^13^C 2D HSQC experiment (Bruker hsqcetgpsisp2.2; phase-sensitive gradient-edited 2D HSQC using adiabatic pulse sequences for inversion and refocusing) was used to collect the main data (Kupče and Freeman, 2007). The HSQC experiments were acquired from 11.5 to −0.5 ppm (12 ppm spectral width) in F2 (^1^H) with 3,448 data points (acquisition time, 200 ms) and 215 to –5 ppm (220 ppm spectral width) in F1 (^13^C) with 618 increments (F1 acquisition time, 8.0 ms) of 8 scans with a 1 s interscan delay (D1); the d24 delay was 0.89 ms (1/8J, *J* = 140 Hz). The total acquisition time for a sample was 1 h 42 min. The spectra were processed using Gaussian apodization (GB = 0.001, LB = −0.5) in F2 and squared cosine-bell in F1; linear prediction was not applied. Volume-integration of contours in HSQC plots was carried out using TopSpin 4.3 (Mac version) software and the data are uncorrected.

### Microscopy

For fluorescence microscopy ^13^C stems embedded in LR White resin were sectioned in the transverse plane using glass knives on a Leica Ultracut microtome at a thickness of 2 µm. Sections were examined by widefield fluorescence after mounting in 50% glycerol in phosphate buffer at pH 9 with blue excitation and green emission to examine the general anatomy and the lignin distribution by autofluorescence (Donaldson, 2013, 2020). Samples of compression wood from a mature tree were sectioned with a sledge microtome at a thickness of 25 µm in the transverse plane and were examined by confocal microscopy (Leica SP5) using sequential excitation at 355 nm (400–500 nm emission) and 488 nm (500–600 nm emission) to visualise G-lignin (blue) and H-lignin (green) by autofluorescence (Donaldson et al., 2010; Donaldson, 2020). To localise galactan, ^13^C samples embedded in LR White resin were sectioned at a thickness of 700 nm using a diamond knife on a Leica Ultracut microtome and immunolabelled with LM5 primary antibody (Kerafast) and Goat anti-Rat secondary antibody labelled with Alexa 633 (Invitrogen) according to standard protocols (Donaldson et al., 2018; Donaldson, 2022). Sections were counterstained with 1% safranine for 5 min and examined by confocal microscopy (Leica SP5) after air drying and mounting in immersion oil. Confocal fluorescence was performed using excitation at 561 and 633 nm with emission at 579–613 (safranine) and 650–790 nm (LM5 epitope). In this case safranine stains whole cell walls (Bond et al., 2008).

### Gas chromatography–mass spectrometry: extraction and analysis

Wood samples (compression and opposite) were collected, debarked, cut into small pieces (1×2 mm) and cryoground with liquid nitrogen. The powdery material (250 mg) was placed in 5 mL of dichloromethane (DCM) and sonicated (150 min). The DCM was removed using a Pasteur pipette and passed through a Na_2_SO_4_ drying tube. An aliquot (1 mL) was then transferred to a GC-MS vial for analysis. The DCM extracts were treated in a similar way as in Patel et al (Patel et al., 2024). To each sample an internal standard (50 µL) was added. The internal standard consisted of 9,10-dibromoanthracene (25 mg) in pyridine (25 mL). The hydroxy groups were converted to trimethylsilyl (TMS) ethers by derivatisation with 50 µL of *N,O*-bis(trimethylsilyl)trifluoroacetamide and trimethylchlorosilane (BSTFA: TMCS, 99:1) followed by heating for 1 h at 70°C before being immediately analysed by GC-MS. Samples were analysed by being injected (1 µL) onto an Agilent 7890B gas chromatograph coupled to a 5977 C single-quad mass spectrometer. An HP-Ultra 2 column (50 m × 200 µm × 0.33 µm) was used as the stationary phase and ultra-high-purity helium (99.999%) was used as the mobile phase at 1 mL min^–1^. A 280°C injection temperature and 300°C interface temperature was used with the following temperature program: 40°C start, ramped at 6°C min^–1^ to 300°C, and held at 300°C for 30 min. The MS source was set at 250°C and the quadrupole set at 150°C with a scanning range of 35–600 m/z and a solvent delay of 13.50 min. Data analysis used Agilent’s Masshunter software (version 10). Compounds were identified by comparing retention times plus mass spectra with reference standards along with NIST14 and in-house mass spectral libraries. Results for each compound are reported as a relative percentage of the entire area of all the peaks in the chromatogram. Results do not reflect relative amounts in the sample but relative amounts in the chromatogram.

### Statistical analysis

Statistical analysis was performed using R (4.2.1). T-tests and 1-way ANOVA were performed if data met their requirements. Otherwise, the non-parametric Wilcoxon Rank Sum and Signed Rank test and Kruskal-Wallis rank sum test were performed, respectively.

### Thermo-porosity by differential scanning calorimetry

Compression, opposite and normal wood sections were cut into ca. 1 × 4 mm pieces with a razor blade then soaked in miliQ water over night. Next day the small wood pieces were cut into 2–3 pieces with a total weight close to 10 mg. The material was placed in an aluminium pan and sealed. Water thermoporosimetry was based on the Gibbs–Thomson effect in which water crystals located in porous structures require more energy to melt due to the pore surface energy. When approximated to perfect cylinders, the diameter of the pores can be calculated from their melting points based on the Equation 1 (Park et al., 2006; Zauer et al., 2014; Majda et al., 2017).


(1) $$\text{D}=4\text{T}0\text{γ cos}(\text{θ})/\left(\left({\text{T}}_{\text{m}}–{\text{T}}_{0}\right)\right)\text{ρHf})$$

Where:

D = diameter of the pore in m; T_m_ = depressed freezing temperature in K;

and

T_0_ = 273.15 K; γ = 12.1 mJ m^−2^; θ = 180°; ρ = 1000 kg m^−3^; Hf = 334 J g^−1^

Thermoporosimetry using DSC is based on a series of arbitrarily chosen isothermal steps (Park et al., 2006; Maloney, 2015; Bourdon et al., 2023). The water melting enthalpy occurring at each isotherm is used to calculate the portion of water retained in the pore within a calculated diameter range. Thermal analysis of the stems was carried out on a Discovery DSC using T-zero hermetic aluminium pans (TA instrument, New Castle, Delaware, USA). The pan containing ~7–12 mg of wood was cooled from room temperature to -30°C following the sequence presented in **Supplementary Table S3**. Samples were soaked overnight under vacuum in milli-Q water, then lightly dabbed with a cotton fabric prior to being sealed in the DSC hermetic pans. The total water content of the fully saturated sample was measured after the DSC test by puncturing the pans and placing them in an oven at 103°C for at least 2 h. The isotherm temperatures were adjusted during the calculation of the corresponding pore diameters by measuring the melting point of milli-Q water (alone) at a ramp of 1°C/min. Experiments were carried out in triplicates and averaged (using three different samples). Results were interpreted with TA Trios v5.1.

### Wide Angle X-Ray scattering and X-Ray diffraction

#### Synchrotron parameters

The *Pinus radiata* wood samples (2-mm-thick stem section) were collected and sealed immediately in Kapton tape. The Synchrotron experiment was conducted within 4 days. The beam energy was set to 15 keV (0.8266 Å) and the camera length was 693.9 mm. One-second shots were collected on a Pilatus3S 2M detector (1475 x 1679 pixels) (Dectris, Switzerland) with a pixel resolution of 172 x 172 µm. Four diffractograms were collected per sample in a 2 × 2 matrix.

Fitting assumed, for simplicity, only a cellulose Iβ structure (Gjönnes et al., 1958). It was possible to assign peaks associated with cellulose *d*(1-10) and *d*(110), modelled as a single peak, cellulose *d*(200), and water. The water peak was attributed based on the work of Hura et al. (Hura et al., 2003). Changes to the crystal packing were determined by the position of the *d*(200) peak maxima and the minimum crystal domain sizes, *L*(200), were calculated using Equation 2 (Scherrer equation):


(2) $$L_{200}=K\lambda /\beta_{\left(200\right)}cos\theta_{\left(200\right)}$$

where K is a crystal geometry parameter, approximated as 0.94 (Murdock, 1930), λ is the X-ray wavelength, β is the width at half peak height, and θ is the Bragg angle.

#### X-Ray diffraction analysis

X-ray scattergrams (32-bit, 1475×1679 pixels) were processed using ImageJ (1.54f). Calibrated images (in q values) of the signal intensity were converted to polar coordinates (radius = q) for processing in R (4.2.1). Data were summarised for q to 3 decimal places and smoothened by rolling mean. A baseline for the q data was determined by fitting a convex hull to the signal intensity versus q plot. The q range and baseline were interactively adjusted to avoid peak truncation. Deflection points in the intensity v q plot were determined by finding peaks in the 2^nd^ derivative. The position of the deflection points was used to inform the deconvolution. Peak deconvolution was performed using the spect_em_pvmm function (Pseudo-Voigt mixture model), EMpeaksR package (Matsumura et al., 2023). _EMpeaksR: Conducting the Peak Fitting Based on the EM Algorithm_. R package version 0.3.1, https://CRAN.R-project.org/package=EMpeaksR). The q range and number of peaks and their initial positions were standardised for all samples processed.

#### Alcohol-insoluble residue preparation

The normal, opposite, and compression wood samples were hand sectioned and cryo-ground for homogenisation. Alcohol-insoluble residue (AIR) was prepared by washing the homogenised woody material with 100% (v/v) ethanol, twice with chloroform:methanol (2:1 v/v), followed by successive washes with 65% (v/v), 80% (v/v), and 100% (v/v) ethanol. The remaining pellet was air dried.

#### Polysaccharide analysis using carbohydrate gel electrophoresis

Hemicelluloses were extracted by treating 50 mg of each AIR preparation with 4 M NaOH for 1 h at room temperature before pH neutralisation at pH 6.0 (adjusted with 1 M HCl). This was followed by a desalting with a PD-10 Sepharose column and elution with 3.5 mL of 50 mM ammonium acetate buffer pH 5.5. Subsequently, 250 µL of eluate were digested with 3 µL of Megazymes GH11 (CAS Number: 9025-57-4) overnight at room temperature. The PACE gel was prepared and ran as described in Goubet et al. (Goubet et al., 2002).

#### Monosaccharide analysis

Monosaccharide compositional analysis was performed as previously described (Liszka et al., 2023). In brief, 1 mg AIR was hydrolysed with 2 M TFA for 2 h at 120°C. Released monosaccharides were derivatised with 3-methyl-1-phenyl-2-pyrazoline-5-one (PMP) to enable detection in UV-VIS and separated on a Synergi 4 µm Fusion-RP 80 Å, LC Column 250 × 4.6 mm manufactured by Phenomenex. The column was mounted on an Agilent Technologies 1260 Infinity II HPLC System. Separation was performed isocratically with an 18:82 (v:v) mix of acetonitrile and 0.1 M phosphate buffer pH 6.6 at a flow rate of 1 mL/min. Monosaccharide annotation was performed by comparing the elution time to that of a standard in which the following sugars were separated: d-mannose, d-ribose, l-rhamnose, d-galacturonic acid, d-glucose, d-galactose, d-xylose, l-arabinose and l-fucose. Integrated signal intensity was used to quantify the molar percent of monosaccharides in each sample.

## Results

### Growing ^13^C labelled conifer secondary cell wall containing compression wood

After growing for six months in the ^13^CO_2_-rich atmosphere the bent pines were taken out for analysis (**Supplementary Figure S1A**). To ensure that the material analysed by ss-NMR was indeed compression wood, a debarked disc of one of the ^13^C-labelled pine stems (6–7 mm diameter) was sectioned and macroscopically imaged (**Supplementary Figure S1B**). The disc upper part was pale (opposite wood) while the lower part had a clearly visible red hue typical of compression wood. Closer observation performed using a confocal microscope capturing cell wall autofluorescence confirmed that the opposite wood part displayed typical features of normal pine secondary cell walls featuring hexagonal cells (**Supplementary Figure S1C**). The lower, reddish part of the stem has all the typical features of severe compression wood: enlarged intercellular space, thicker and rounder tracheids, presence of helical cavities in the lumen, and stronger lignin autofluorescence (**Supplementary Figure S1D**). To confirm that this material contained (1→4)-β-galactan, which is a typical marker of the compression wood, we also performed a labelling using the LM5 monoclonal antibody that specifically recognises (1→4)-β-galactan epitopes (Andersen et al., 2016). The image clearly shows, as expected, a specific binding of the LM5 antibody in the outer S2L region (**Supplementary Figure S1E**). Lastly, **Supplementary Figure S1F** reveals the presence of H-lignin throughout the S2L using its specific autofluorescence emission spectra. We therefore have confirmation that the ^13^C-labelled material contains both compression and opposite wood SCW.

### Differences in composition and molecular arrangements revealed by solid-state NMR

**Figure 1:**
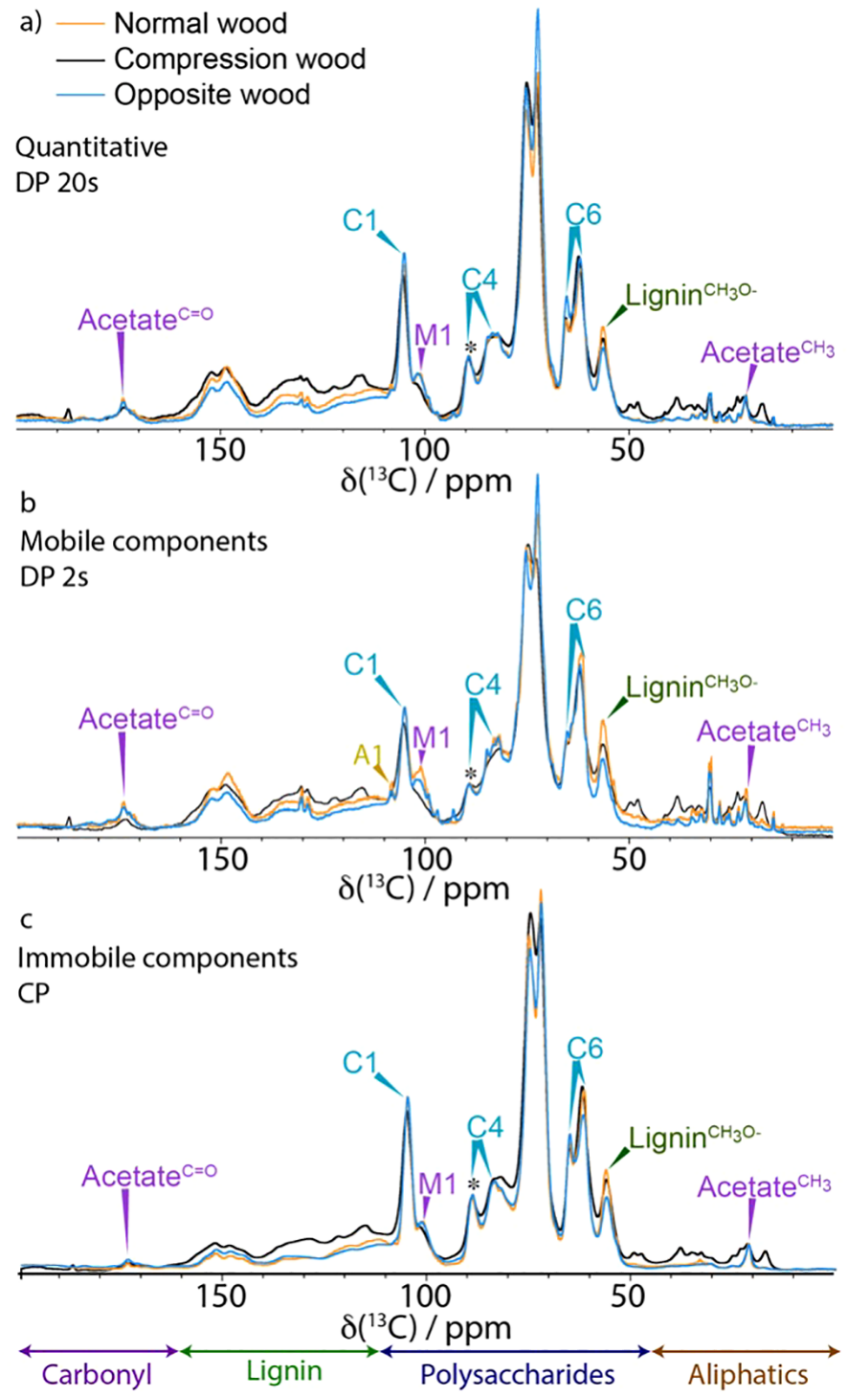
A comparison of 1D ^13^C NMR spectrum of normal wood (orange), compression wood (black) and opposite wood (blue). **(a)** Quantitative (DP 20s), **(b)** Mobile (DP 2 s) and **(c)** Immobile (CP). Spectra have been normalized to the C4^1^ cellulose peak at 89 ppm (marked with *) and were recorded at a ^13^C Larmor frequency of 150.7 MHz and a MAS frequency of 12 kHz.

To gain an overview of the compositional variations and major molecular environmental changes for the different types of pine SCWs, we first performed one-dimensional (1D) ^13^C ss-NMR experiments. A 1D cross-polarisation (CP) experiment as well as two 1D direct polarisation (DP) experiments with different recycle delays were acquired from all three woods. The CP experiment preferentially enhances signals from relatively immobile components within the cell wall whereas DP with a 2 s recycle delay emphasises the mobile components. The 20 s DP experiment gives a near quantitative spectrum of all the components in each sample. These spectra from never-dried normal, opposite, and compression pine wood are shown, overlaid, in **Figure 1** with an expanded view of the polysaccharide region in **Supplementary Figure S2**. The peaks are assigned using previous reports and can be separated into four major regions: carbonyls, lignin, polysaccharides, and aliphatics (Wang et al., 2012; Dupree et al., 2015; Gao et al., 2023).

The quantitative 1D ^13^C ss-NMR spectra (**Figure 1a**) shows that there is substantially more lignin in the compression wood (visible between 110 ppm and 160 ppm) compared to normal and opposite wood. Enhanced lignin intensity is particularly visible in this region at ~115, 122 and 156 ppm indicating a difference in the lignin composition. This observation is consistent with previous microscopic and biochemical characterisation of compression wood having a higher lignin content with a substantial amount of the usually minor H-type lignin. To further investigate the lignin compositional changes a CP Non-Quaternary Suppression (NQS) experiment was used to suppress the signal from carbons with directly attached protons. This experiment removes most of the signal from the carbohydrates leaving the lignin quaternary peaks (110–160 ppm) as shown in **Supplementary Figure S3**. A comparison of the NQS spectra reveals that the lignin peak positions are slightly shifted, and their ratios differ between compression wood and normal pine confirming the altered nature of the lignin in compression wood. In the opposite wood there is a slight reduction of lignin content relative to normal wood, but its composition was similar.

**Figure 1b** also reveals the presence of several new peaks in the compression wood spectrum in the carbonyl (160–180 ppm) and in the aliphatic (50–15 ppm) regions. The analysis of GC-MS of these extractives, summarised in **Supplementary Figure S4A**, revealed that compression wood contained larger amounts of terpenes (3-carene and β-pinene), fatty acids (palmitic and stearic acid), and phenols (isovanillin). Opposite wood contained more resin acids, the individual components of which are presented in **Supplementary Figure S4B**. The 1D CP in **Figures 1C** (and **S2C**), shows that the relatively immobile components in normal and opposite wood look similar except for the cellulose C6 domain 2 which is larger despite no notable changes in the corresponding C4 region.

Two well-known major compositional particularities of compression wood are that it can contain up to 10% w/w of galactan (Nanayakkara, 2007) and that H-units can be up to 30% of the lignin (Timell, 1986). The large increase of galactose seen in the monosaccharide analysis of compression wood confirms the presence of (1→4)-β-galactan in our sample (**Supplementary Figure S5**). It also revealed a small increase of galactose content in opposite wood consistent with the change of amplitude seen near 62 ppm in the **Figure 1c**. Our monosaccharide analysis (**Supplementary Figure S5**) confirms that opposite wood contains more galactan than normal wood. These results agree with Chavan et al., who demonstrated that opposite wood contains (1→4)-β-galactans but in a much lower proportion than in compression wood (Chavan et al., 2015). For the solution state NMR experiment, the stem was divided into two parts: the severe compression wood and the remaining part that which contained mild compression wood, normal wood and opposite wood. Solution-state NMR (**Supplementary Figure S6A**) of severe compression wood and the remaining part of the wood (**Supplementary Figure S6B**) also clearly reveals the presence of galactan in both samples. The compositional differences are most strikingly seen in the 2D-difference spectra, **Supplementary Figure S6C** in which the G-lignin peaks were nulled to reveal the H-lignin units most clearly, and in **Supplementary Figure S6D** in which the glucan/xylan peaks were nulled to reveal the Gal peaks more clearly. Although such solution-state HSQC spectra are not quantitative, and freely rotating end-units (of which H-units are disproportionately high) are particularly overquantified, the volume-integrals listed in the yellow boxes in **Supplementary Figures S6A, 6B** reveal the substantial elevation of H-units (by over 3-fold) in the severe compression wood over the remaining part of the wood, and the elevated galactan levels (by some 2.1-fold). Accompanying the changes in the aromatic composition (from an H/G of ~5% in the opposite wood to ~17% in the severe compression wood), the distribution of inter-unit linkage types (**A:B:C**) in the polymers is mildly affected with a slightly lower β-ether unit **A** level and an elevated resinol **C** level consistent with the elevated H-unit content. In addition to the markedly elevated galactan levels in the severe compression wood, solution-state NMR (**Supplementary Figure S6**) easily identifies the presence of lower levels of (1→4)-β-galactans in the remaining wood.

**Figure 2:**
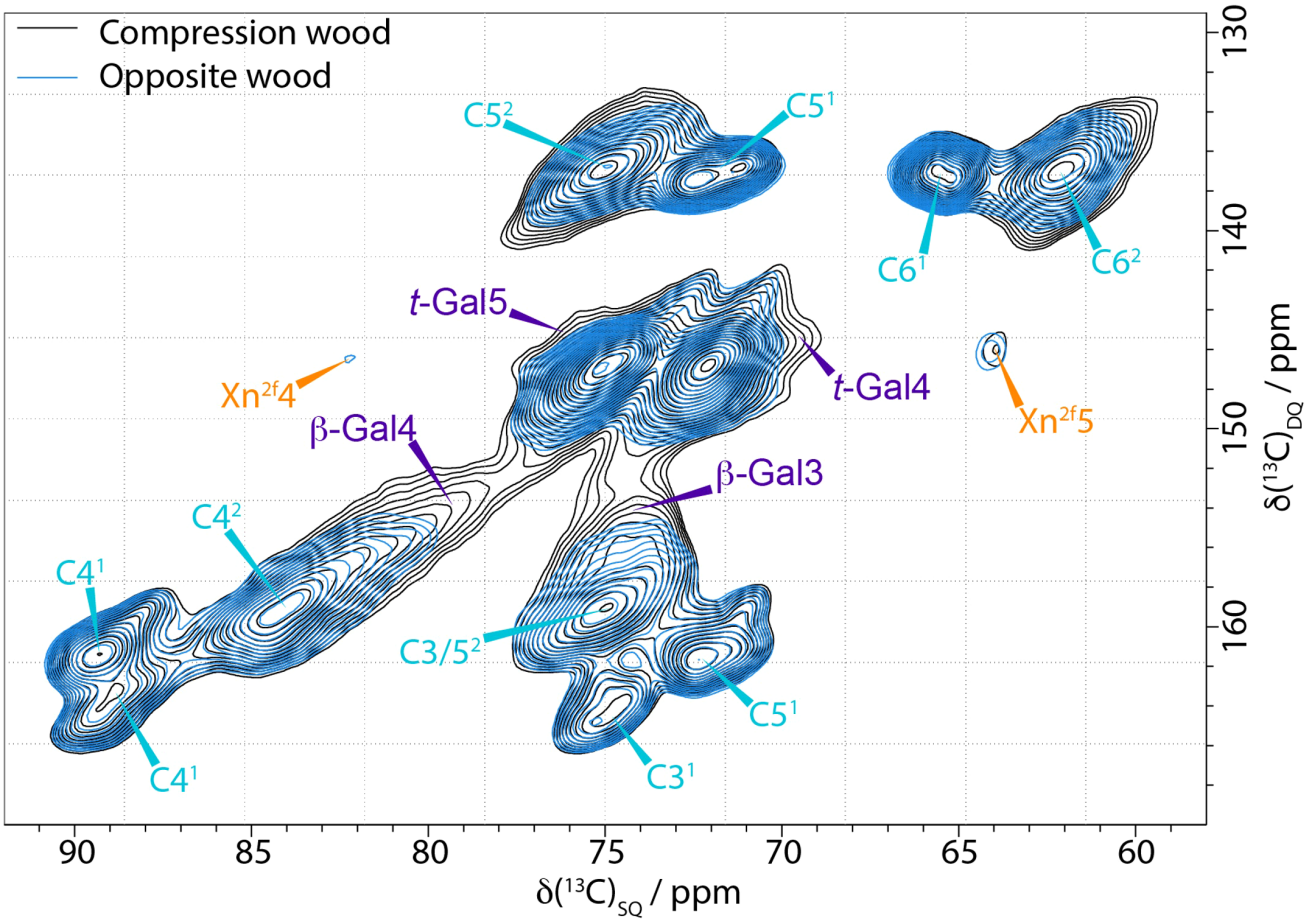
Comparison of the C4-C5−C6 region of ^13^C CP-INADEQUATE NMR spectra of compression wood (black) and opposite wood (blue) normalised to the cellulose C4^1^ peak at 89 ppm. The spectra were recorded at a ^13^C Larmor frequency 150.7 MHz and a MAS frequency of 12 kHz. The spin-echo duration used was 2.2 ms.

In the solid state NMR spectra, all the ^13^C shifts of galactan overlap with those of cellulose. The inability of 1D ss-NMR to reveal the galactan-specific signals means that 2D ss-NMR experiments such as refocussed INADEQUATE or PDSD are needed. **Figure 2** is the comparison of the C4–C6 region of compression and opposite wood CP INADEQUATE spectra. The peaks in an INADEQUATE spectrum arise from directly bonded ^13^C nuclei with the shift on the DQ axis being the sum of the shifts of the carbons that are bonded (see for example the xylan Xn^2f^4 and Xn^2f^5 in **Figure 2**). The spectrum of compression wood is significantly broader than that of opposite wood, so it remains difficult to clearly resolve the galactan peaks. However, there is substantial intensity at ~78.5 ppm and 74.5 ppm (the C4 and C3 shifts of 4-linked galactan respectively) compared to the opposite wood consistent with the presence of increased levels of (1→4)-β-galactan (Gao et al., 2023). There is also intensity at the expected position for the C4 of *t*-Gal at 69.5 ppm visible at the DQ shift corresponding to C4–C5 ~146 ppm.

A DP INADEQUATE experiment with a short recycle delay (2 s) preferentially reveals the relatively more mobile components of the cell wall such as some hemicellulosic and pectic polysaccharides. However, the DP INADEQUATE spectrum of compression wood (**Supplementary Figure S7**), was also very broad further highlighting the lack of mobility of the cell wall components in compression wood. Both the CP and DP INADEQUATE spectra of compression wood display a broad signal at ~78.5 ppm and ~74.5 ppm which is not distinguishable in the opposite wood, further confirming the presence of the (1→4)-β-galactan.The terminal galactan (t-Gal) C4 and C5 shift positions are clearly observable but are not fully resolved in the DP INADEQUATE spectrum.

Although both terminal (*t*-Gal) and (1→4)-β-galactan (β-Gal) are difficult to resolve in the either the CP or DP INADEQUATE spectra they could be clearly identified using a CP PDSD experiment. This experiment relies on the dipole-dipole interaction and thus depends strongly on distance, providing insight into the proximities of the different components in the cell wall. For short mixing times, such as 30 ms, cross peaks are only observed between close carbons, mostly those within the same sugar residue. The C4 shifts of both *t*-Gal and β-Gal are different from cellulose (see **Supplementary Table S4**) and so slices taken at these positions can be compared for opposite and compression wood and the difference should be galactan, if present. **Supplementary Figure S8A** shows slices taken at 69.5 ppm, the *t*-Gal C4 shift and their difference and in **S8B** slices taken at 78.5 ppm, the (1→4)-β-galactan C4 shift. In **Supplementary Figure S8A** all the terminal galactan shifts are clearly visible in the difference as are all the (1→4)-β-galactan shifts in **S8B**. However, the linewidths of the spectra are much larger than that observed in, e.g., *Arabidopsis thaliana* (Gao et al., 2023) indicating that there is considerable disorder in compression wood galactan.

**Figure 3:**
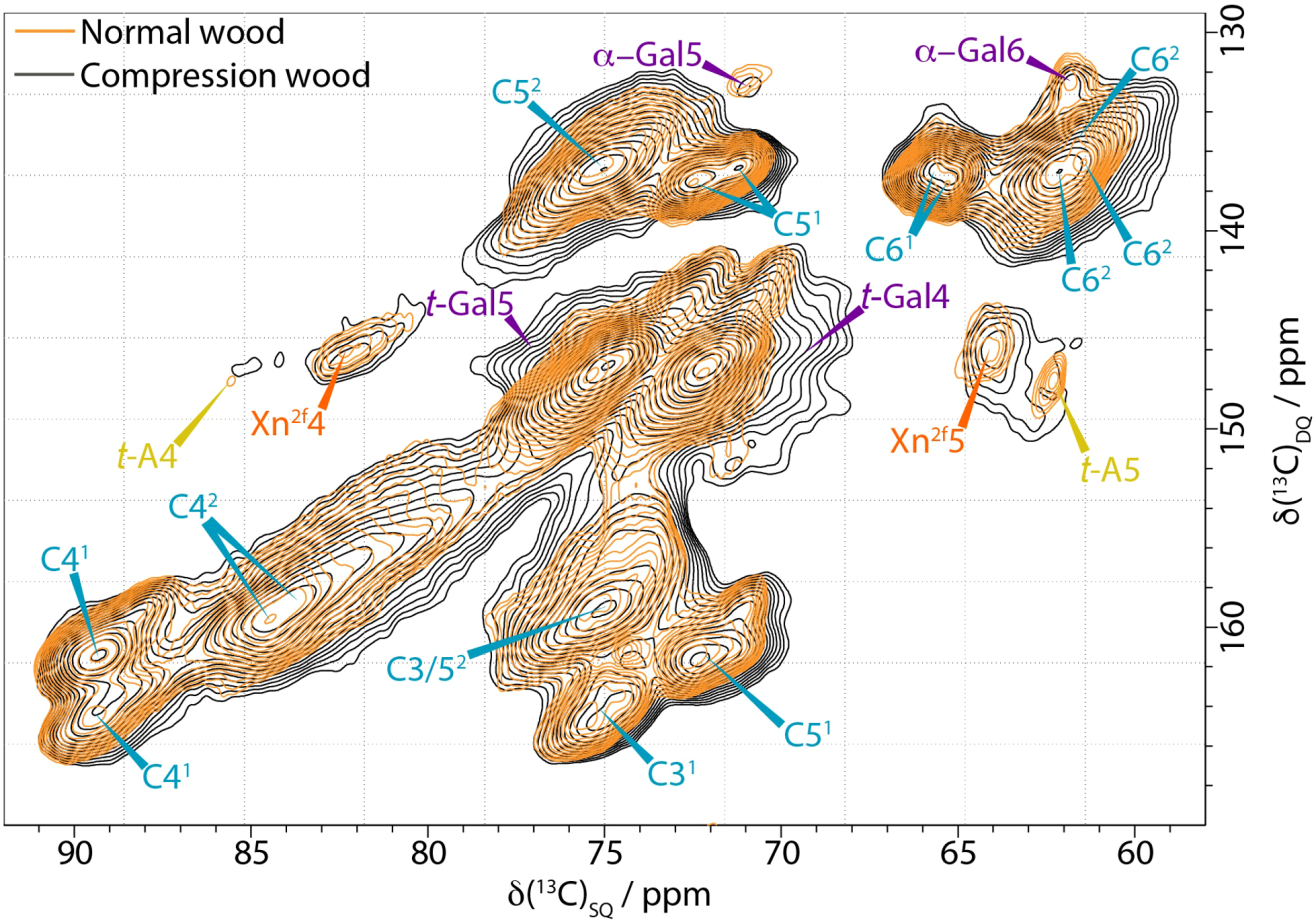
Comparison of the C4-C5−C6 region of ^13^C CP-INADEQUATE NMR spectra of normal wood (orange) and compression wood (black) normalised to the cellulose C4^1^ peak at 89 ppm. The normal wood spectrum was recorded at a ^13^C Larmor frequency of 176.0 MHz and a MAS frequency of 12.5 kHz. The compression wood was recorded at a ^13^C Larmor frequency 150.7 MHz and a MAS frequency of 12 kHz. The spin-echo duration used was 2.2 ms.

**Figure 3** shows a comparison of the cellulose C4-C6 region of the CP INADEQUATE spectra for compression and normal wood. The spectrum of compression wood is significantly broader than that of both normal and opposite wood. Furthermore, the C5 of terminal arabinose, *t-*A5, is no longer clearly visible and the xylan Xn^2f^4 and Xn^2f^5 are significantly broader. Another hemicellulosic side chain on the mannan, α-Gal, also shows some broadening in the compression wood. We know that AGX (arabinoglucuronoxylan) and α-Gal are sensitive to the hydration environment and the broadening observed in compression wood is reminiscent of our previous observations from oven-dried pine (Cresswell et al., 2021). There are no significant differences in the cellulose domain 1 environments of compression and normal wood. However, a significant difference was observed in the cellulose domain 2 peaks particularly in the C5-C6 regions. The three distinct cellulose environments that are observed in the normal, never-dried pine spectrum are no longer distinguishable and there is a large increase in intensity at ~ 61.9 ppm in compression wood. Some of the increase in intensity at 61.9 ppm is due to the C6 peak of galactan; however the loss of resolution from the different cellulose environments in the domain 2 region is also indicative of a reduction of hydration and/or increase in disorder in compression wood compared to normal wood.

**Figure 4:**
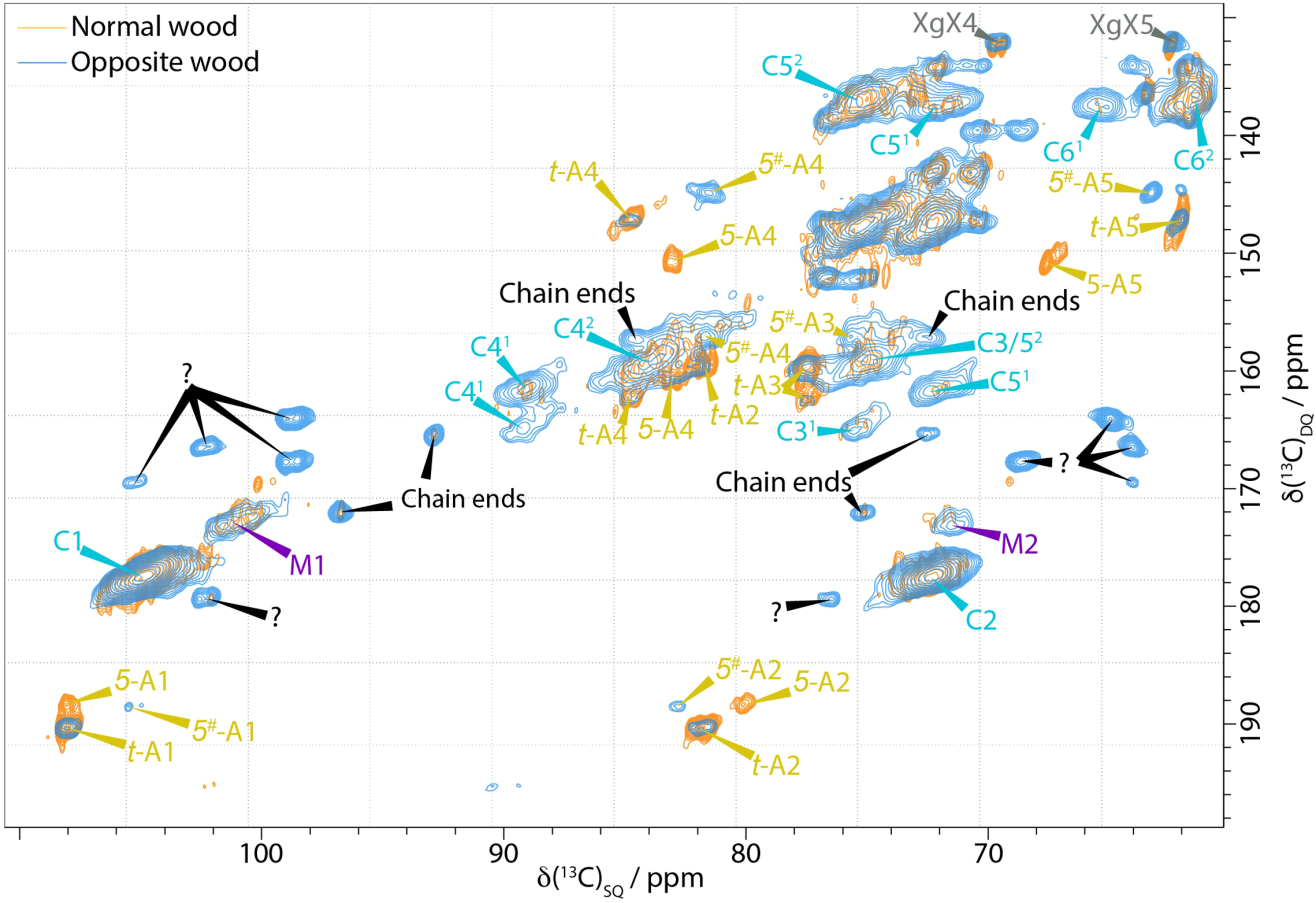
Comparison of the neutral carbohydrate region of a ^13^C DP-INADEQUATE NMR spectra of never-dried pine (orange) and opposite wood (blue). Novel (Unknown, marked with a question mark) mobile components are present in the opposite wood as well as distinct changes in the arabinoses. Spectra were recorded at a ^13^C Larmor frequency of 150.7 MHz and a MAS frequency of 12 kHz.

We have also found that there are distinct differences between opposite wood and normal pine wood. The 2s 1D DP ss-NMR spectrum of the opposite wood shown in **Figure 1b** reveals several mobile components unseen in the normal wood with the chain reducing ends at 97 and 93 ppm being particularly visible. However, there are many overlapping peaks that make it difficult to characterise any further significant differences in the 1D spectrum. The DP INADEQUATE spectrum shown in **Figure 4** reveals more clearly the extent of the differences in the mobile components of the opposite wood and normal pine. The cellulose peaks are broader and more intense (e.g. C6^1^) in opposite wood. There is also a large increase in the amount of chain reducing ends in the opposite wood compared to the normal wood in agreement with the 1D ss-NMR spectra. The spectrum also shows a significant change in the arabinose present in the wood. The 5-linked arabinose in normal wood with a C4 at 83.0 ppm and C5 at 67.6 ppm is no longer present in the opposite wood, whilst there is a different arabinose with C4 and C5 shifts of 81.6 ppm and 63.2 ppm respectively. There is also a set of five unidentified C1-C2 peaks in the DP INADEQUATE that have not been observed in any of our softwood samples including compression wood and that have only been seen in substantial amounts, but not assigned, in primary cell wall materials from Arabidopsis (Phyo et al., 2017).

### Differences in mobility and water accessibility of the SCW components

Both CP and DP 1D ss-NMR spectra of compression wood show significant broadening of many of the polysaccharide components compared to both opposite wood and normal wood (**Figures 1B, C**). The broadening is particularly evident in the M1 peak at 101 ppm and the Xn^2f^4 peak at 82 ppm and in the arabinose sidechains (visible in the CP INADEQUATE in **Figure 3**) indicating increased disorder as well as reduced mobility in compression wood. The increased disorder of the Xn^2f^4 at 82 ppm and the slight shift of the M1 peak by ~0.2 ppm at 101.0 ppm are reminiscent of that observed in oven dried pine (Cresswell et al., 2021). The ^13^C spin lattice relaxation times, T_1_, for compression wood, shown in **Supplementary Table S5**, are significantly longer, apart from the lignin methoxy group, than that of normal wood also indicative of reduced mobility of the cell wall components in compression wood. The relaxation times of opposite wood are intermediate largely falling between those of normal wood and compression wood.

We have previously shown that water is a structuring element of the plant cell wall polymers that influences wood mechanical properties and mobility (Cresswell et al., 2021). It has been reported numerous times that green, never dried, compression wood has a lower water content (~35-25% lower) than normal wood (Timell, 1986). The water content of the compression wood was investigated therefore using 1D ^1^H ss-NMR and X-ray diffraction. The proton spectrum (**Supplementary Figure S9A**) show that the water content of the compression wood sample is around 40% of normal never-dried wood and only double that seen in oven-dried normal pine wood. The ^1^H NMR shift of the water in compression wood is different compared to both opposite wood and normal wood indicating that the water in compression wood is in a different local environment. The X-ray diffraction displays a peak attributable to the water that is exhibiting some degree of order over the time scale of the experiment. We refer to this water as “organized” water. The organized water content was estimated by comparing the integration of the peak intensities from organized water and cellulose (200) (**Supplementary Figure S9B**). **Supplementary Table S6** confirms that compression and opposite wood can be grouped together as they exhibit significantly lower quantities of organized water content than normal wood. These experiments reveal a similar trend to that found from ^1^H NMR with both compression wood and opposite wood having a lower water content than normal wood.

**Figure 5:**
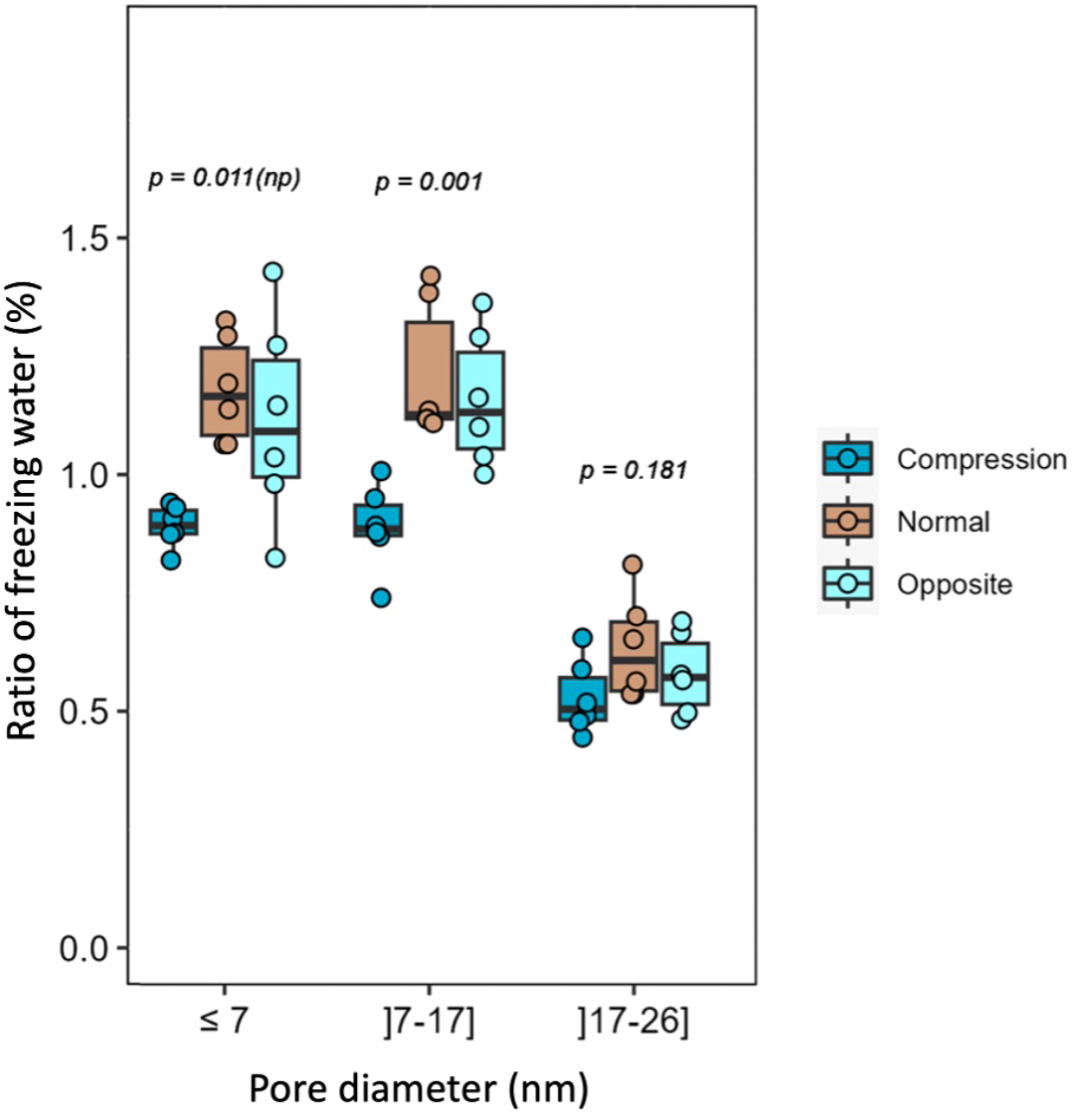
Ratio of freezing water for compression, normal, and opposite wood. DSC thermoporosimetry box plot representing the estimated distribution of pore diameter in association with the ratio of freezing water (in percentage). The error bars indicate the 95% confidence interval. The *p* is the parametric p-value and *np* is the non-parametric p-value.

A comparison of the ^1^H T_2_ relaxation times of the water in different woods reveals that the T_2_ of both compression and opposite wood is very much shorter than that for normal wood (**Supplementary Table S7**). This indicates that there are smaller ‘pools’ of mobile water in the compression wood and opposite wood than in normal wood (Maunu, 2002). The size of these ‘pools’ of bulk water could be related to the pore size in the sample and so their distribution was measured using DSC thermoporosimetry measurements (Bourdon et al., 2023). The results indicate reduced nanoporosity for compression wood compared to normal and opposite wood over the whole range of porosity tested (**Figure 5**). For pores below 7 nm and between 7 and 17 nm the ratio of freezing water is significantly smaller indicating that the volume of the nano-pores in compression wood is reduced by ~30% compared to normal wood. Thus, the reduced nanoporosity and organized water content, together with our NMR results indicate that, in compression wood, both the water molecular mobility and content are significantly reduced. To ensure that the observed amount of water in the structure of the secondary cell wall of compression wood is not a handling artefact, and to potentially improve the spectral resolution, an attempt was made to increase the hydration of the compression wood cell walls. The sample was left in a 100% humidity atmosphere for two weeks and reanalysed. The proton spectrum shown in **Supplementary Figure S10** shows that the water content in the compression wood increased by ~40%. However, the subsequent 1D DP 2s spectrum in **Supplementary Figure S11A** shows little change in either the resolution or the mobility of the compression wood. Although the cell wall of the as-received compression wood is less hydrated than normal wood, the additional water therefore had no major effect on the mobility of the SCW polymers in compression wood indicating they are less water accessible than in normal wood. However, the peaks attributable to dehydroabietic acid (**Supplementary Figure S11B**) were smaller after the hydration possibly indicating that some has gone into solution and could therefore not be detected in our experiments.

**Figure 6:**
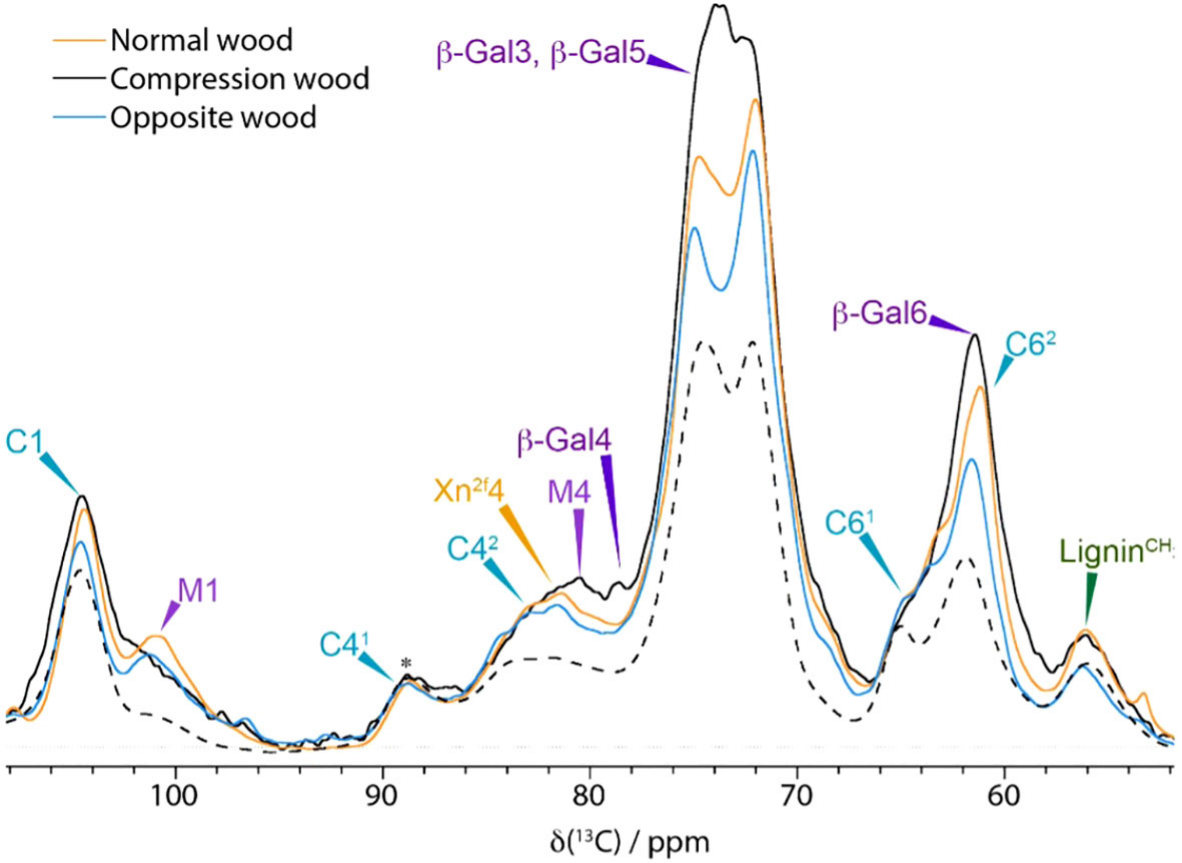
Comparison of 1D water edited spectra with a proton filter of 1 ms and a short diffusion time of 1 ms of Compression wood (black), Normal wood (orange), Opposite wood (blue) and a standard 1D CP spectrum of compression wood (black dashed). Spectra were recorded at a ^13^C Larmor frequency of 150.7 MHz and a MAS frequency of 12 kHz.

To investigate the location of the mobile pools of water in the SCW a 1D water-edited ss-NMR experiment was performed. In this experiment, the cell wall components that are in close proximity to the more mobile pools of water have their signals enhanced as they build up faster than those further away. A comparison of the normal CP spectrum with a water-edited spectrum acquired with diffusion time of 1 ms (and normalised to the cellulose C4^1^ at 89 ppm) for all three types of wood is shown in **Figure 6**. At this short diffusion time the 61.8 ppm peak in the compression wood spectrum, which is likely to have a contribution from galactan C6, is enhanced in the water-edited experiment relative to the normal CP spectrum. The ratio of the ~72–75 ppm peak heights is inverted in the water-edited spectrum of the compression wood, almost certainly due to the influence of the galactan C2,3,5, whose shifts lie in this region, of the spectrum, i.e., galactan is close to water. Further information is provided from the signal build-up curves that are shown for a few selected peaks in **Supplementary Figure S12**. Mannan (101 ppm) is the closest to water in all types of wood. In compression wood, the second fastest component to build-up is the Gal6/C6^2^ peak at ~62 ppm. In opposite and normal wood the C6^2^ cellulose (62 ppm) and 2-fold xylan (Xn^2f^4 at ~82 ppm) are the second fastest components building-up at similar rates. Altogether these experiments start to a build the picture of compression wood cell walls as having less water, and in which the mobile water is close to an external layer comprised of mannan and galactan whereas xylan and cellulose are further away.

### Proximity differences in compression wood *vs.* normal wood

**Figure 7:**
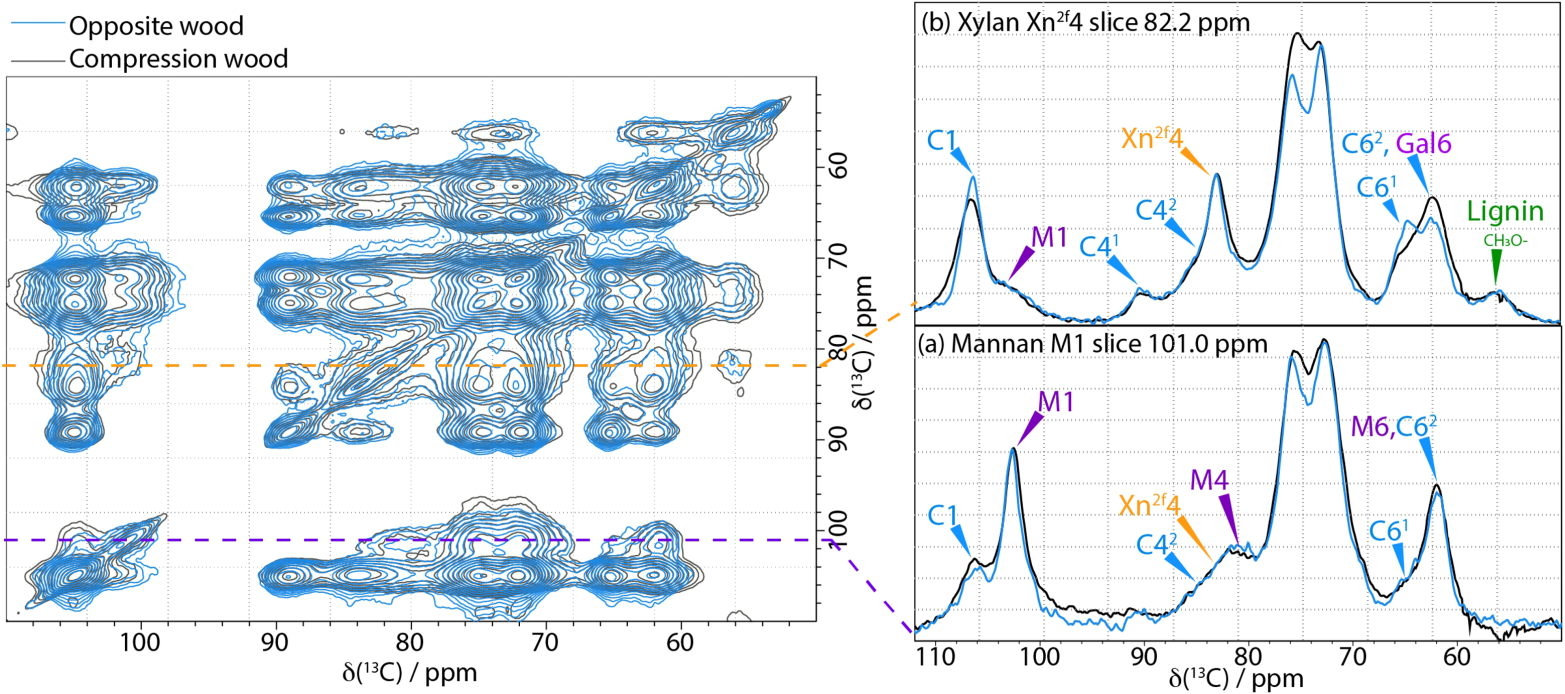
Left-hand side: A comparison of 400 ms ^13^C CP-PDSD NMR spectra of Opposite wood (blue) and Compression wood (black) normalised to the self-peak of cellulose C1 at 105 ppm. Right-hand side: Slices are taken from the CP PDSD spectrum to highlight the key differences in hemicelluloses between never-dried pine and compression wood: **(a)** slice at the mannan M1 shift, 101 ppm; **(b)** slice at the xylan X4 shift, 82.2 ppm; Spectra were recorded at a ^13^C Larmor frequency of 150.7 MHz and a MAS frequency of 12 kHz.

To investigate if any of the proximities of the components within the secondary cell wall have altered due to the presence of galactan in compression wood, 2D CP PDSD spectra were acquired with a mixing time of 400 ms to probe length-scales of up to 5–8 Å. **Figure 7** is an overlay of the neutral carbohydrate region of the 400 ms CP PDSD spectra of compression wood and opposite wood with selected slices highlighting differences in the proximities of the main hemicelluloses, xylan and mannan, **Figure 7a** is a comparison of the slices from the M1 of mannan at 101.0 ppm and indicates that there is very little change in the proximity of mannan to cellulose and xylan in the compression wood compared to opposite wood. This is different from that observed in oven-dried wood (Cresswell et al., 2021) despite their both having reduced hydration.

For the three types of wood, no obvious changes in the type and ratio of xylan oligosaccharide released by a GH11 digestion was observed (**Supplementary Figure S13**). This indicates that the ratio of predominant xylan pattern (with evenly distributed side-chains) and minor xylan pattern (with consecutive glucuronic acid substitution) is maintained (Busse-Wicher et al., 2016; Martínez-Abad et al., 2017). The GUX1 and GUX2 activity is therefore likely to be similar in normal, opposite and compression wood (Lyczakowski et al., 2021). **Figure 7b** shows the slices taken at 82.2 ppm (normalised to the xylan Xn^2f^4 peak) which reveal that most of the cellulose cross-peaks (C1, C4^1^, C6^1^) are smaller in the compression wood than in opposite wood. There is also a significant change in the line shape in the slice between 68–80 ppm and the peak at 61.9 ppm is more prominent. These changes are all likely due to the presence of galactan in compression wood. They indicate that xylan and galactan are close to each other and could suggest xylan and galactan have a specific interaction in compression wood.

A slice of the 400 ms CP PDSD spectrum of compression wood taken at the H-lignin carbon shift of 115.5 ppm shows the H-lignin environments (**Supplementary Figure S14**). It unequivocally indicates that H-lignin is sufficiently close (within <5 Å) to the cellulose C1 and/or Xn^2f^1 for a cross peak to be observed as well as weak cross peaks to ~187 ppm and some carbons in the aliphatic region. These are shown more clearly in the 2D spectrum of this region (**Supplementary Figure S15**) where many of the connections to the peak at ~187 ppm are visible allowing the assignment of the narrow peaks seen here and in the 1D slice to dehydroabietic acid with no other fatty or resin acids being observed. The presence of cross peaks in the 115.5 ppm slice indicates that dehydroabietic acid is relatively close to H-lignin.

### Cellulose crystal hydrogen bonding and minimum crystal domain size

As changes in the matrix composition and molecular arrangement of compression wood have been observed, we investigated their influence on microfibril arrangement and cellulose structure itself using X-ray diffraction. The d-spacing of the (200) cellulose peak (*d_200_*) was found to be 3.917, 3.933 and 3.952 Å for normal, opposite and compression wood, respectively (**Supplementary Figure S16**). While the difference in d-spacing between normal and opposite wood is small compression wood exhibited a significantly larger d-spacing. This indicates an increased distance in the intersheet spacing of the cellulose molecules in compression wood (**Supplementary Table S8**). The minimum crystal domain size (*L_200_*) was calculated to be 19.59, 20.37 and 20.87 Å for the normal, opposite and compression wood, respectively (**Supplementary Figure S17**). Kruskel-Wallis and pair-wise statistical analysis comparisons showed significant differences between normal and opposite as well as between normal and compression wood (**Supplementary Table S9**). This indicates that normal wood, *L_200_* and therefore the cellulose microfibrillar crystallite width is slightly but significantly smaller than that of opposite and compression wood.

## Discussion

Reaction wood has been described as the “muscles” of plants (Gorshkova et al., 2018). More specifically in conifer compression wood has an economic relevance as it is an undesirable trait for lumber (Hassegawa et al., 2020). The use of ^13^C-labelled softwood has allowed the development of secondary cell wall models (Dupree et al., 2015; Terrett et al., 2019; Kang et al., 2019; Addison et al., 2024) and highlighted the importance of water as a structural element (Cresswell et al., 2021), with the production of ^13^C-labelled compression and opposite wood we can use solid state NMR to further investigate the differences of these specialised tissues compared to normal softwood. We have investigated compression, opposite and normal wood composition, water status and the proximities between the various polymers and their proximity to water. Taken together the data allow us to gain a better understanding of compression wood and the basis of its biomechanical properties.

### Composition

Our solid-state NMR results revealed the different compositional changes that were expected such as the increase in lignin content, enhancement of H-lignin levels, and a change in extractive composition (**Figure 1**). However, although the presence of galactan was confirmed by immunolocalization (**Supplementary Figure S1D**), monosaccharide analysis (**Supplementary Figure S5**), and solution state NMR (**Supplementary Figure S8**), it is less clearly resolved using solid-state NMR as the shifts of both β-1–4 and *t-*galactan are largely overlapping with shifts of cellulose (**Figure 2**).

Extractives represent around 1.5 wt.% of *Pinus radiata* sapwood (Uprichard and Lloyd, 1980). Resin acids contain the tricyclic diterpenoids defence metabolites such as dehydroabietic and abietic acids (Costa et al., 2016). These hydrophobic extractives are known to modulate the water binding in wood (Choong and Achmadi, 1991) and to lower the equilibrium moisture content (Fredriksson et al., 2023). Compared to opposite wood, our GC-MS results showed that compression wood had an increase in extractives amounts (terpene, fatty acid and phenolic compounds) (**Supplementary Figure S4**). Although the diterpenoid content was lower overall in compression wood their signal in the NQS spectrum is enhanced (**Supplementary Figure S3**). This indicates that the dehydroabietic acids are in a different form in compression and opposite wood. They are mobile (likely solubilised) in opposite wood and immobilised in compression wood, probably as part of a larger macromolecular complex. The immobilisation of the dehydroabietic acid could be explained by its proximity to H-lignin (**Supplementary Figure S11**). This H-lignin-to resin acid association could be a playing a key role in modulating the properties of the S2L layer of the highly lignified compression wood. These antibacterial lignin/diterpenoid complexes could also play a role in increased hydrophobicity, reduced molecular mobility, reduction of accessibility [reduced nanoporosity (**Figure 5**)], and the high resistance to bacterial decay (Kim et al., 2022). The decrease of resin acid peak intensity observed after two weeks of hydration (**Supplementary Figure S11**), is possibly due to the lability of these compounds. For example, it is well known that palustric acid, one of the main resin acids (**Supplementary Figure S4**), isomerises readily (Wallis and Weame, 1997; Zender et al., 1994; Morales et al., 1992).

### Molecular architecture and water

The CP/DP INADEQUATE spectra (**Figures 2**–**4** and **S6**) reveal some unexpected differences between opposite, normal, and compression wood. Opposite wood has generally been considered to be chemically identical to normal wood. However, the DP INADEQUATE ss-NMR spectra (**Figure 4**), which predominantly reveal the mobile components, show that the intensity of the cellulose shifts in opposite wood is much larger than in normal wood. Together with an increase of the signal from chain reducing ends, this suggests a greater mobility of the cellulose of opposite wood compared to normal softwood. The DP INADEQUATE also reveals differences in the mobile arabinose present in the opposite wood as well as several unidentified mobile components not previously observed in softwood. This is the first time that a chemical difference between normal and opposite wood has been demonstrated. The CP INADEQUATE spectrum, which predominantly shows the relatively immobile components, of compression wood (**Figures 2**, **3**) is broader than that of normal wood, which in turn is broader than that of opposite wood. Further confirming that the polymers in opposite wood have an increased mobility compared to normal wood whereas for compression wood the overall molecular mobility is reduced and there is significantly more disorder. In cell walls the increase in polymer disorder/reduction in mobility is a phenomenon possibly enhanced due to a reduction in hydration (Ha et al., 1997). Our previous work demonstrated that water must be considered as a key player for the polymer arrangement within native cell walls (Cresswell et al., 2021). We therefore investigated the overall hydration of the different wood types and showed that cell wall water content in compression wood is much reduced (**Supplementary Figure S9**). This is consistent with our pore size estimation showing that compression wood nanoporosity is also reduced (**Figure 5**) and is likely to be correlated to a lower fibre saturation point of never-dried compression wood (Timell, 1986; Simpson and Barton, 1991). Altogether, our observations are in agreement with the recent finding showing that cell wall pore sizes decrease with a decrease of moisture content (Youssefian et al., 2017; Sun et al., 2024).

Recently, three types of water have been described in pine softwood: the “free” water in the lumen, and two types of “hard-to-remove” water within the cell walls. The hard-to-remove water present within the cell walls consists of “bound” water that is part of the hydrated matrix and “highly-bound” water whose self-diffusion is constrained by its entrapment between the microfibril surface and its direct environment (Jarvis, 2018; Li and Ma, 2022; Salem et al., 2023). These NMR observations are in agreement with the interpretation of electron tomography experiments and small-angle neutron scattering suggesting the potential for a significant fraction of water molecules to reside and diffuse along spaces between individual cellulose microfibrils (Penttilä et al., 2021; Fernando et al., 2023). Our measurements of the water spin-spin relaxation time (^1^H T_2_), shows that it is greatly reduced in compression wood (**Supplementary Table S7**) implying that the pools of weakly bound water are smaller in compression wood compared to normal wood again corroborating the reduction of nanoporosity of compression wood (**Figure 5**). The higher proportion of bound and highly-bound water in compression wood also fits with previous observation that compression wood is slower to dry (Wimmer and Johansson, 2013). Furthermore, an increase of cell wall water content in compression wood does not change the molecular mobility (**Supplementary Figures S10, S11**). Our observations of porosity and hydration reduction in compression wood agree with the conclusions from previous publications in which compression wood is described as less permeable, with a lower diffusion coefficient and hydraulic conductivity than normal wood both in longitudinal and radial directions (Mayr and Cochard, 2003; Tarmian and Perre, 2009; Tarmian et al., 2012). These characteristics mean that compression wood acts as a barrier to moisture migration (Martin et al., 2021).

As in never-dried normal wood the hemicelluloses acGGM and AGX are closely associated with cellulose (**Figure 7**) in both compression and opposite wood. The only noticeable difference is that a portion of the 2-fold-screw xylan might be more remote from the cellulose microfibril or alternatively have a specific interaction with the galactan in compression wood. The latter hypothesis could be related to the recent discovery that RG-I is covalently linked to AGX in softwood primary cell walls (Makarova and Shakhmatov, 2024).

Our *d_200_* values (3.92–3.95 Å) from X-ray diffraction (**Supplementary Figure S16**) are slightly lower than the ones reported for the softwood Sugi (3.96 Å), and the hardwoods birch (3.94 Å) and cherry (3.96 Å) (Abe and Yamamoto, 2006; Thomas et al., 2014). However, it is known that d-spacings (distances between crystallographic planes) of cellulose crystallites vary with cell wall hydration (Abe and Yamamoto, 2005; Zabler et al., 2010). Our samples were freshly collected from young plantlets and never dried whereas previous studies used dried and then rehydrated mature wood. Note that, we have previously shown for normal wood, dehydration of cell walls leads to irreversible changes at the molecular scale (Cresswell et al., 2021). The higher (200) d-spacing of compression wood suggests that that the cellulose chains are more loosely packed. This could be the result of a reduction in moisture content (Yamamoto et al., 2009; Paajanen et al., 2022) and/or the decrease in AGX and acGGM hemicellulose content relative to the cellulose.

The *L_200_* values from our pine wood samples (19.6–20.9 Å) are a third lower than those usually found in the literature (25–36 Å for spruce) (Andersson et al., 2003). Previous measurements were performed on dried mature wood whereas, as described above, our samples were freshly harvested from young plantlets and still in a never-dried state. It is also likely that differences from other published L values arise from instrument setup and parameters used, approaches to peak deconvolution, and overlapping peaks. These were kept constant during this set of experiments. Thus even though our values are different from those usually found in the literature, the comparison between them will be valid. The fact that the minimum crystallite size of opposite and compression wood were similar but different to normal wood reinforces the idea that opposite wood is different from normal wood. The change in crystal size, increase in polymer mobility, and increase in oxidising and reducing chain ends in opposite wood could be the result of this side of the stem being slightly under tension. At the beginning of the 20^th^ century opposite wood was named *tension wood* (Timell, 1986). The softwood secondary cell walls would see two opposite adaptation mechanisms to mechanical stress. The compressed part would have a reduced molecular mobility whereas the opposite part under tension would see an increase in molecular mobility, with both mechanisms increasing the microfibrillar width. The fact that we can find differences at the molecular scale between opposite and normal wood is reminiscent of the differences found between late and early wood (Liszka et al., 2023). This highlights the fact that wood is not a homogenous composite but that the polymers and their molecular arrangements are diverse continuum within the same tree depending on the maturity, position, season of biosynthesis, etc.

### Galactan and biomechanics

Our results suggest that compression wood β-(1→4)-galactan is disordered, and well mixed within the cellulose microfibril’s close environment. The long and linear chains of galactan are therefore likely to form a three-dimensional network. This network would impair the water diffusion as well as generate and maintain the stress in gymnosperm compression wood. In angiosperms, tension wood G-layer porosity increases during maturation (Chang et al., 2015). It is proposed that this phenomenon is created by the swelling of the hydrated matrix stretching the cellulose trellis network to generate the maturation tensile stress. Being the opposite adaptation, we would suggest the high microfibril angle of compression wood combined with increased lignification and our observed dehydration and porosity reduction is part of the mechanism leading to locked compressive stresses at the cellular level during the maturation of the softwood secondary cell walls. During compression wood formation calcium is required (Du and Yamamoto, 2003), and calcium-related genes are up-regulated (Li et al., 2013; Sato et al., 2014). This divalent cation could also be involved in locking the compressive stress, by crosslinking the galactan network in a similar fashion as in the pectin egg-box model (Cao et al., 2020). Lastly, compression wood and opposite wood both have galactan but we showed that their hemicellulose molecular mobility is different, suggesting that the galactan chain influence on the wall properties might not be the same in both wood types.

### Model

Altogether, our observations are sufficient to allow us to build a model of compression wood molecular architecture to compare with normal wood. In this model the cellulose microfibrils and their direct surroundings are similar. However, the microfibril is surrounded by less mannan and less xylan but far more galactan randomly distributed along the microfibril (**Supplementary Figures S8A, B**). In addition, we find that in all three types of wood mannan is the closest to the weakly bound water, followed by the galactan, then xylan and cellulose (**Supplementary Figure S12**).

**Figure 8:**
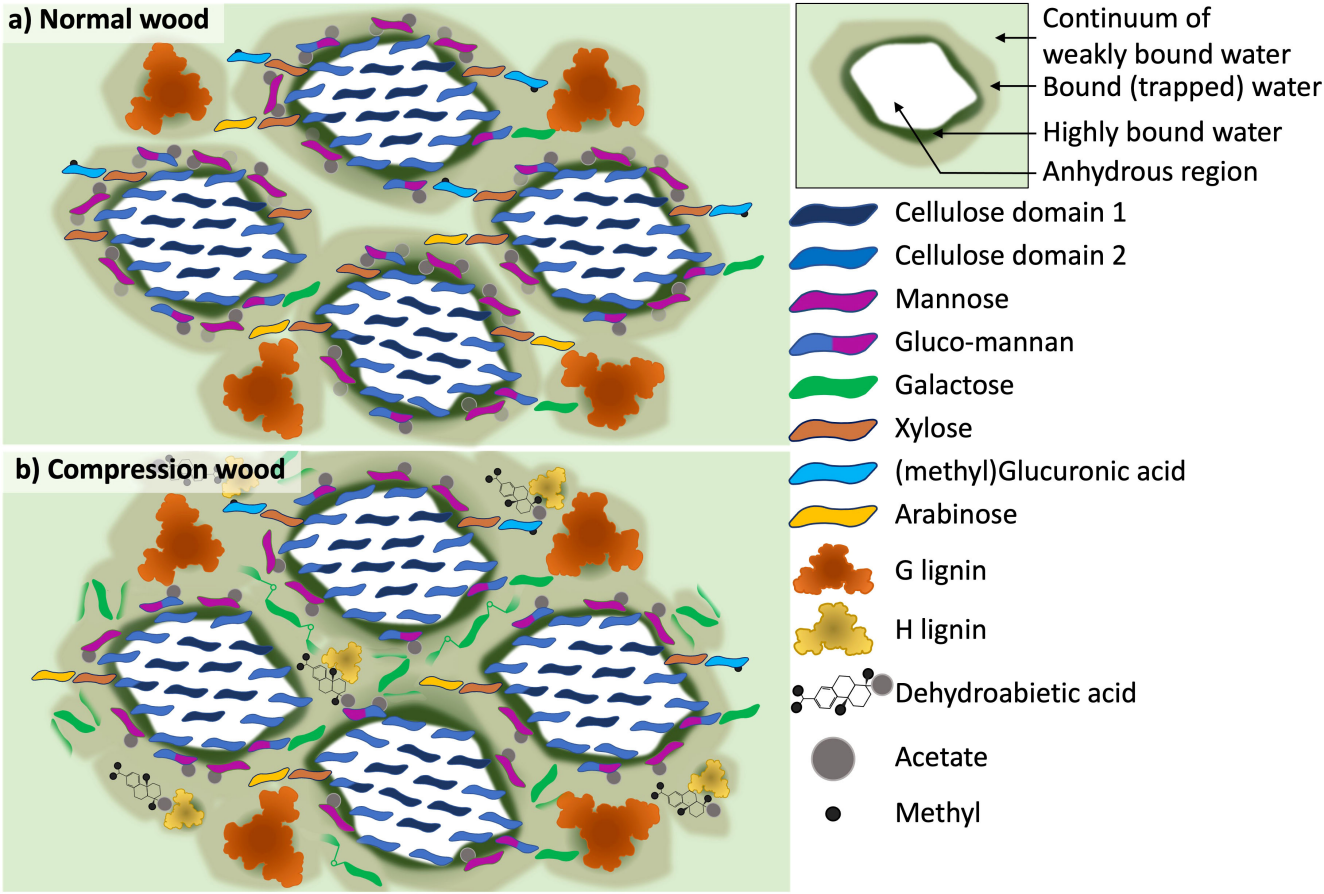
Schematic models which show the differences in the cell wall of **(a)** normal wood and **(b)** compression wood as interpreted from the experimental solid-state NMR, XRD and compositional analysis. The models show a transverse section of a cellulose microfibril with a 2-3-4-4-3–2 habit surrounded by the hemicellulose and lignin matrix. **(a)** Is a four microfibril illustration of our previous never-dried softwood model (Cresswell et al., 2021), where both galactoglucomannan (GGM) and xylan are interacting with the cellulose microfibril surface and there is a range of bound water present in the cell wall. **(b)** the compression wood model shows the addition of disordered galactan, H-lignin and dehydroabietic acid to the cell wall. The model also illustrates the significant reduction of weakly bound water in the compression wood where it was found that the majority of water present is in a more bound/trapped state.

Using these elements we have adapted our never-dried normal softwood model to compression wood. For this model we represented four microfibrils with a spatial distribution based on the molecular model of Paajanen (Paajanen et al., 2022). One of our aims was to represent the distribution of the different pools of bound water surrounding the microfibrils. The difference between normal and compression wood are (**Figure 8**):

- In compression wood, the cellulose microfibrils are slightly closer to each other which is an effect that was observed in molecular modelling (Paajanen et al., 2022). The spacing between the cellulose molecules (*d_200_*) is increased as observed (**Supplementary Figure S16**).
- Compression wood has a reduction in acetate, mannan, xylan, arabinose and methylated glucuronic acid content.
- Compression wood is enriched with H-lignin which is represented as being smaller than G-lignin because lignin enriched in H-units have a lower degree of polymerization (Mottiar et al., 2016).
- The dehydroabietic acids are added close to the H-lignin as these two molecules were detected as being in proximity.
- The galactan molecules are oriented randomly compared to the microfibril direction indicating the relative disorder of the galactan compared to other hemicelluloses such as mannan. The proximity of galactan to xylan was shown in our results but as discussed there are several different interpretations for how the galactan could maintain this close proximity so this not fully illustrated in our current model.
- To represent the continuum of different pools of bound water we used three shades of green. The dark green represent a monolayer of highly bound water forming the water hydration shell surrounding the anhydrous cellulose microfibril (Salem et al., 2023). The light green in the background represents the weakly-bound water and the brownish green represents the bound or trapped water associated with the matrix. The compression wood model displays an increase of bound water and a reduction of the weakly bound water pool. The reduction in weakly bound water is correlated with a reduction in nanoporosity.

## Conclusion

This work takes advantage of recent advances in the understanding of normal never-dried and dried softwood (Terrett et al., 2019 and Cresswell et al., 2021) to decipher the macromolecular arrangement of cell wall polymers in compression and opposite wood. The findings highlight the differences between compression, normal and opposite wood at the molecular architectural level. Interestingly, the molecular mobility within compression wood is decreased whereas it is increased in opposite wood. Compression wood also exhibits a high proportion of highly-bound water in its cell wall which is likely the result of the long galactan chains that are well mixed and disorganised at the molecular scale. We revealed the immobilisation of the dehydroabietic acid likely associated with the H-lignin in compression wood. The labile associations between resin acid extractives and phenolic compounds are potentially important at shaping the hydro-mechanical properties of wood and timber. These observations point toward the complexity of the softwood secondary cell wall where the matrix composition and organisation is likely to be a continuum of variations. Our work is therefore a continuation of the Münch observation that pertinently described normal wood as being a transitional form between opposite wood and compression wood (Münch, 1938). Indeed, although normal and opposite wood are chemically very similar they are not equivalent (Timell, 1986).

## Data Availability

The datasets presented in this study can be found in online repositories. The names of the repository/repositories and accession number(s) can be found below: The Raw solid state NMR Data can be accessed via the following link: https://wrap.warwick.ac.uk/189736/.
