# Supplementary material for: The molecular architecture distinctions between compression, opposite and normal wood of Pinus radiata
Source: Front Plant Sci. 2025 May 13;16:1576928. doi: 10.3389/fpls.2025.1576928 (PMC12106396; doi:10.3389/fpls.2025.1576928)
Supplement: Supplementary file 1 [file DataSheet1.pdf]

Supplementary Information

Contents

Supplementary Figures

Figure S1: Images of the ^{13}C labelled pine stem and cell walls containing compression wood.

Figure S2: A comparison of the carbohydrate region of 1D ^{13}C NMR spectrum of normal wood (orange), compression wood (black) and opposite wood (blue).

Figure S3: Comparison of the lignin region of the CP-NQS NMR spectra.

Figure S4: a) Extraction of terpenes, fatty and resin acids and phenols of compression and opposite wood. b) breakdown of the resin acids. The axis is % of total extract with DCM and analysed via GC-MS.

Figure S5: Monosaccharide analysis of normal, opposite and compression wood.

Figure S6: Comparison HSQC solution-state ^{13}C NMR spectra of compression wood and opposite wood.

Figure S7: Comparison of the C4-C5–C6 region of ^{13}C DP-INADEQUATE NMR spectra of compression wood (black) and opposite wood (blue) normalised to the cellulose $C4^1$ peak at 89 ppm.

Figure S8: Comparison of slices from the 30 ms CP PDSD spectra of compression wood (black) and opposite wood (blue) and their difference (mauve).

Figure S9: a) Comparison of 1D ^1H echo spectra of never dried pine (orange), compression wood (black), opposite wood (blue) and oven dried wood (mauve). b) Water/cellulose peak intensity ratio measured from X-Ray diffraction experiment. c) Example of X-Ray scattergram of *Pinus radiata* normal wood. d) Diffracted intensity showing the deconvolved peaks based on the intensity of the scattering pattern.

Figure S10: Comparison of the 1D ^1H spectra of compression wood (black) vs rehydrated compression wood (blue) after 14 days in a 100% humidity atmosphere.

Figure S11: a) Comparison of 1D DP 2s spectra of compression wood (black) vs rehydrated compression wood (blue).

Figure S12: Comparison of build-up curves of a water-edited experiment for never dried pine, compression wood and opposite wood showing that mannan (blue) is closest to water in all woods.

Figure S13: Polysaccharide Analysis Using Carbohydrate Gel Electrophoresis (PACE) of hemicelluloses extracted from normal, opposite and compression wood of *Pinus radiata* AIR cell walls.

Figure S14: Comparison of the H lignin peak at 115.5 ppm slice of the 400 ms CP PDSD spectra of compression wood (black) and opposite wood (blue).

Figure S15: The aliphatic region of 400 ms ^{13}C CP-PDSD NMR spectra of compression wood showing the cross peaks for the dehydroabietic acid.

Figure S16: Calculated d-spacing for the (200) diffraction peak.

Figure S17: Calculated lateral crystal minimum domain size based on the (200) diffraction.

Supplementary Tables

Table S1: Structural and supramolecular differences found in the normal to severe compression wood continuum.

Table S2: Chemical differences found in the normal to severe compression wood continuum.

Table S3: Subsequent ramp/isothermal steps.

Table S4: The NMR shifts of galactan identified in compression wood compared with those of domain 2 cellulose.

Table S5: ^{13}C solid-state NMR T_1 relaxation times for all three wood samples.

Table S6: Mean value of the water/cellulose XRD peak intensity ratio and the statistical analysis.

Table S7: Comparison of hydration of all wood samples and ^{1}H NMR T_2 relaxation times for all wood samples fit to a single exponential.

Table S8: Mean d_{200} value with statistical analysis.

Table S9: Mean minimal lateral crystal domain size L_{200} with statistical analysis

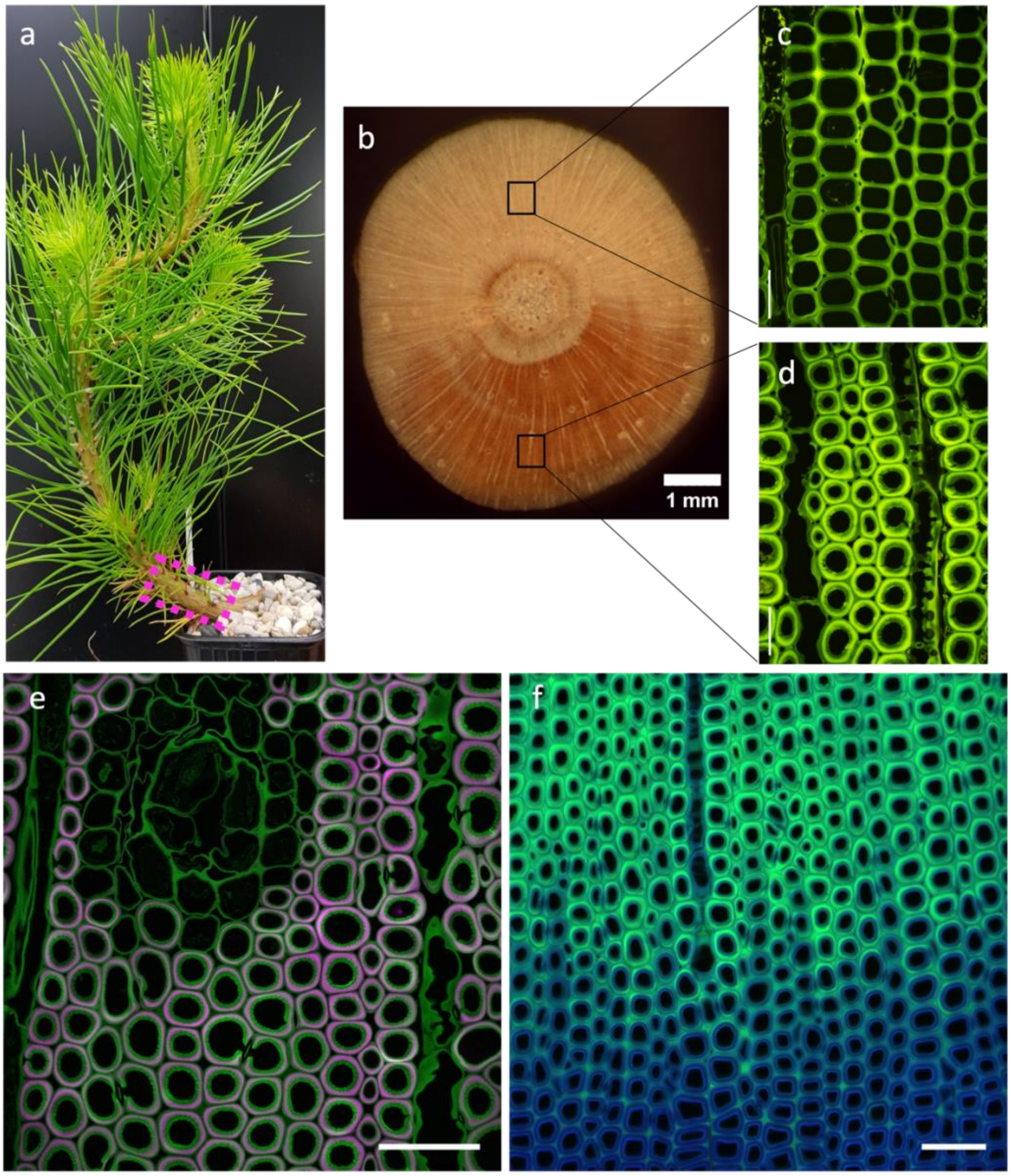

Figure S1: Images of the ^{13}***C labelled pine stem and cell walls containing compression wood.*** *a) Picture of one of the* ^{13}*C labelled pine producing compression wood. The pink dotted square shows the area where the compression wood was collected. b) Macroscopic image of a whole transversal section of the* ^{13}*C labelled stem. c) Widefield autofluorescence image of opposite wood (scale bar = 30 µm). d) Widefield autofluorescence image of compression wood (scale bar = 30 µm). e) Confocal image colocalization of Galactan. LM5 labelling (magenta) counterstained with safranine (green). f) Confocal image of normal and compression wood autofluorescence reflecting the likely distribution of H-lignin (green) and G-lignin (blue). Scalebars = 50 µm.*

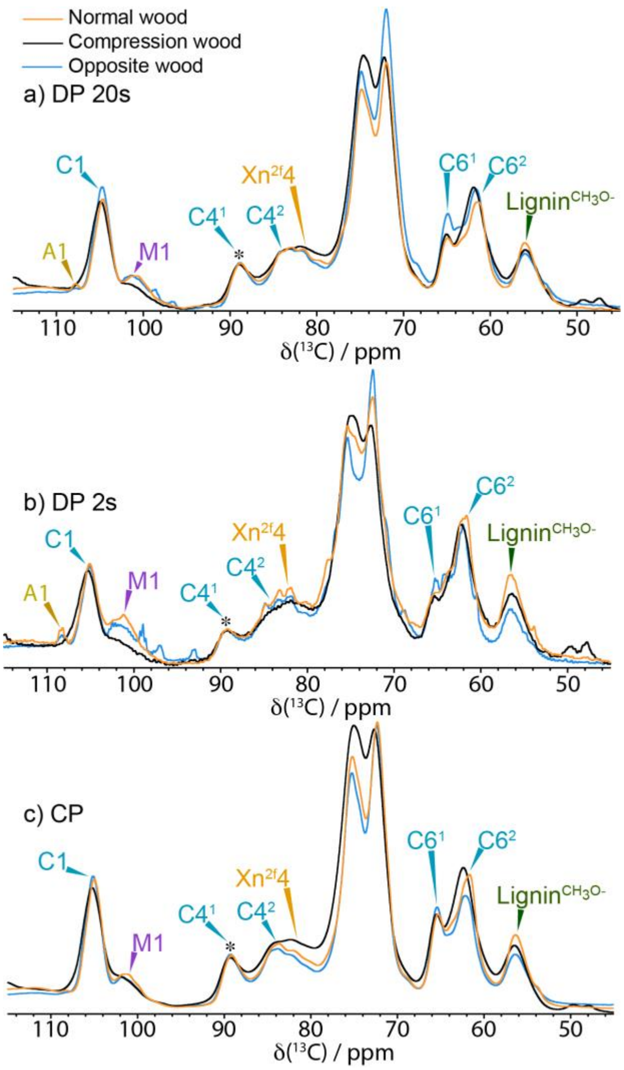

Figure S2*: A comparison of the carbohydrate region of 1D ^{13}C NMR spectrum of normal wood (orange), compression wood (black) and opposite wood (blue). a) Quantitative (DP 20s), b) Mobile (DP 2 s) and c) Immobile (CP). Spectra have been normalized to the $C4^1$ cellulose peak at 89 ppm (marked with *) and were recorded at a ^{13}C Larmor frequency of 150.7 MHz and a MAS frequency of 12 kHz.*

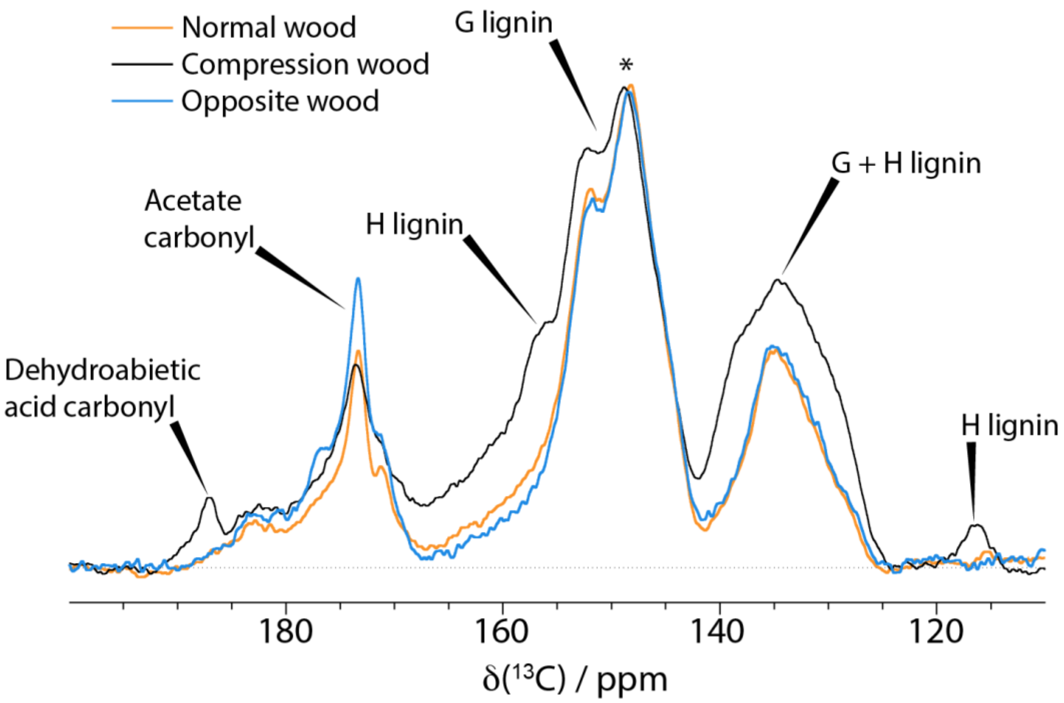

Figure S3: *Comparison of the lignin region of the CP-NQS spectra with a dephasing delay of 45 us of never-dried pine (orange) v compression wood (black) v opposite wood (blue) normalised to the G lignin peak at 150 ppm marked by an asterisk.*

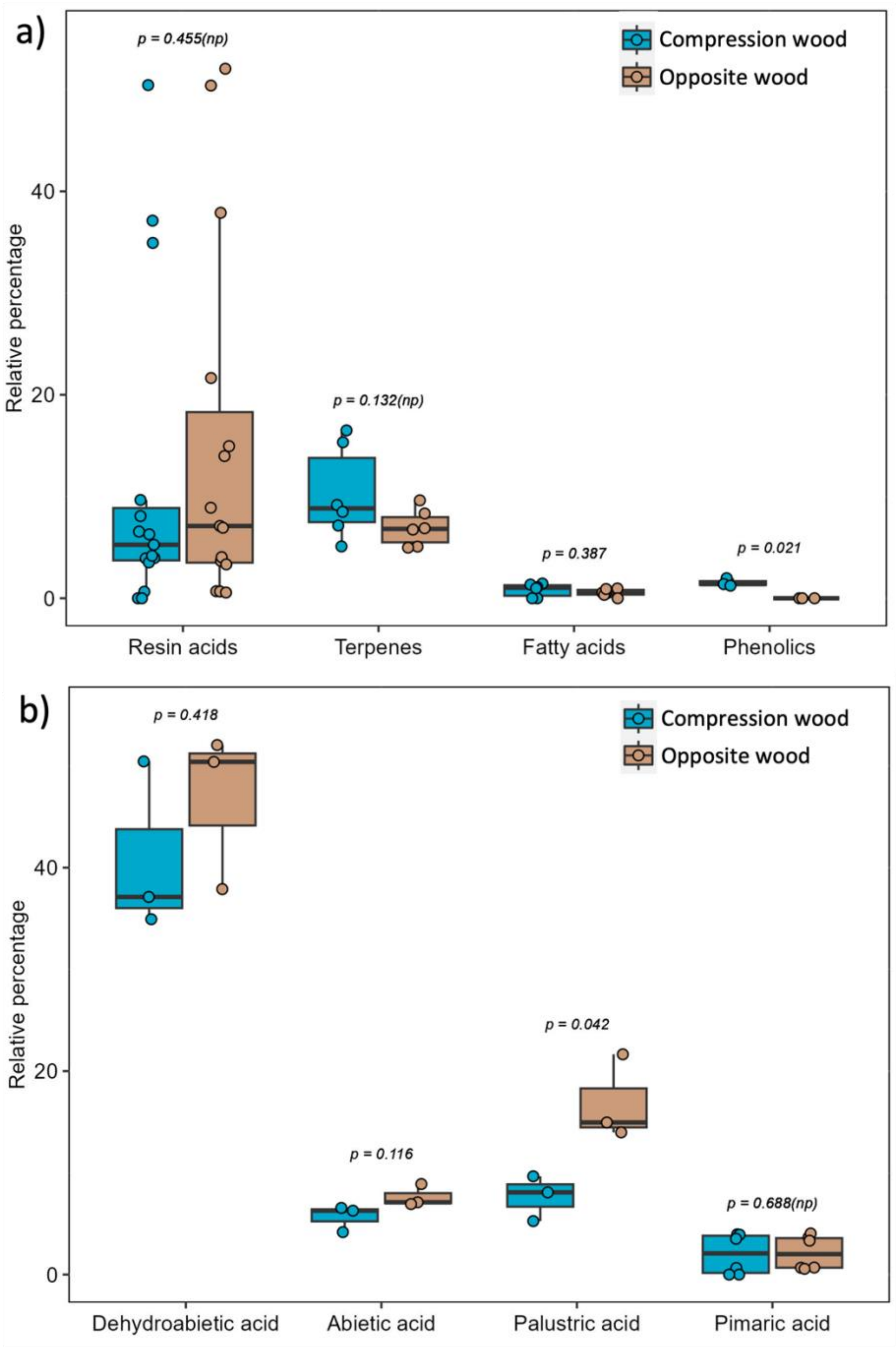

Figure S4: *a) Extraction of terpenes, fatty and resin acids and phenols of compression and opposite wood. b) breakdown of the resin acids. The axis is % of total extract with DCM and analysed via GC-MS. The p is the parametric p-value and np is the non-parametric p-value.*

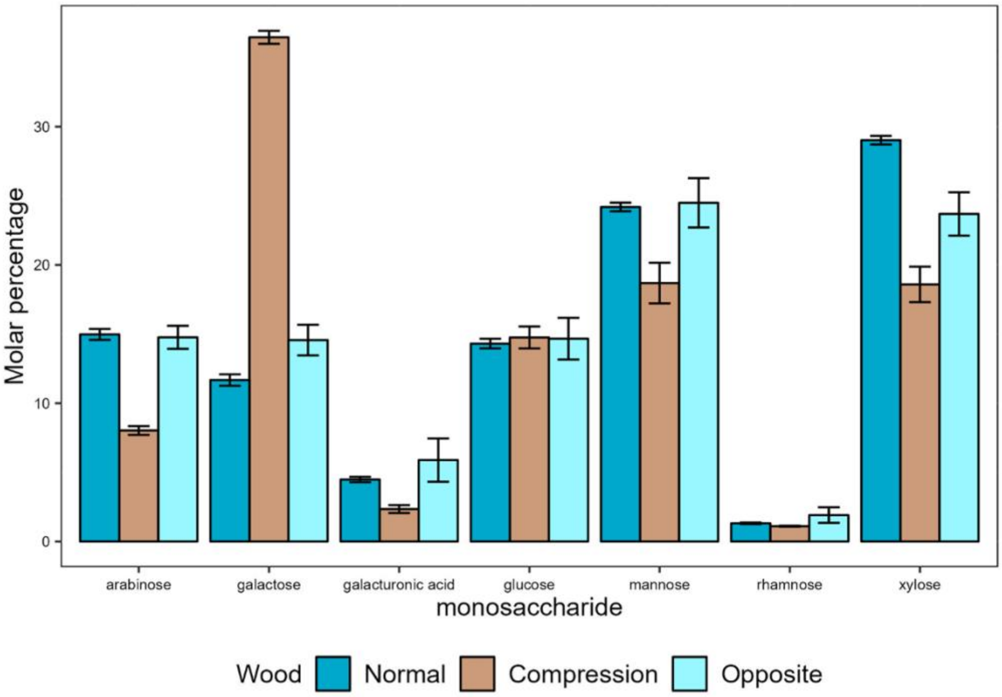

Figure S5*: monosaccharide analysis of normal, opposite and compression wood*.

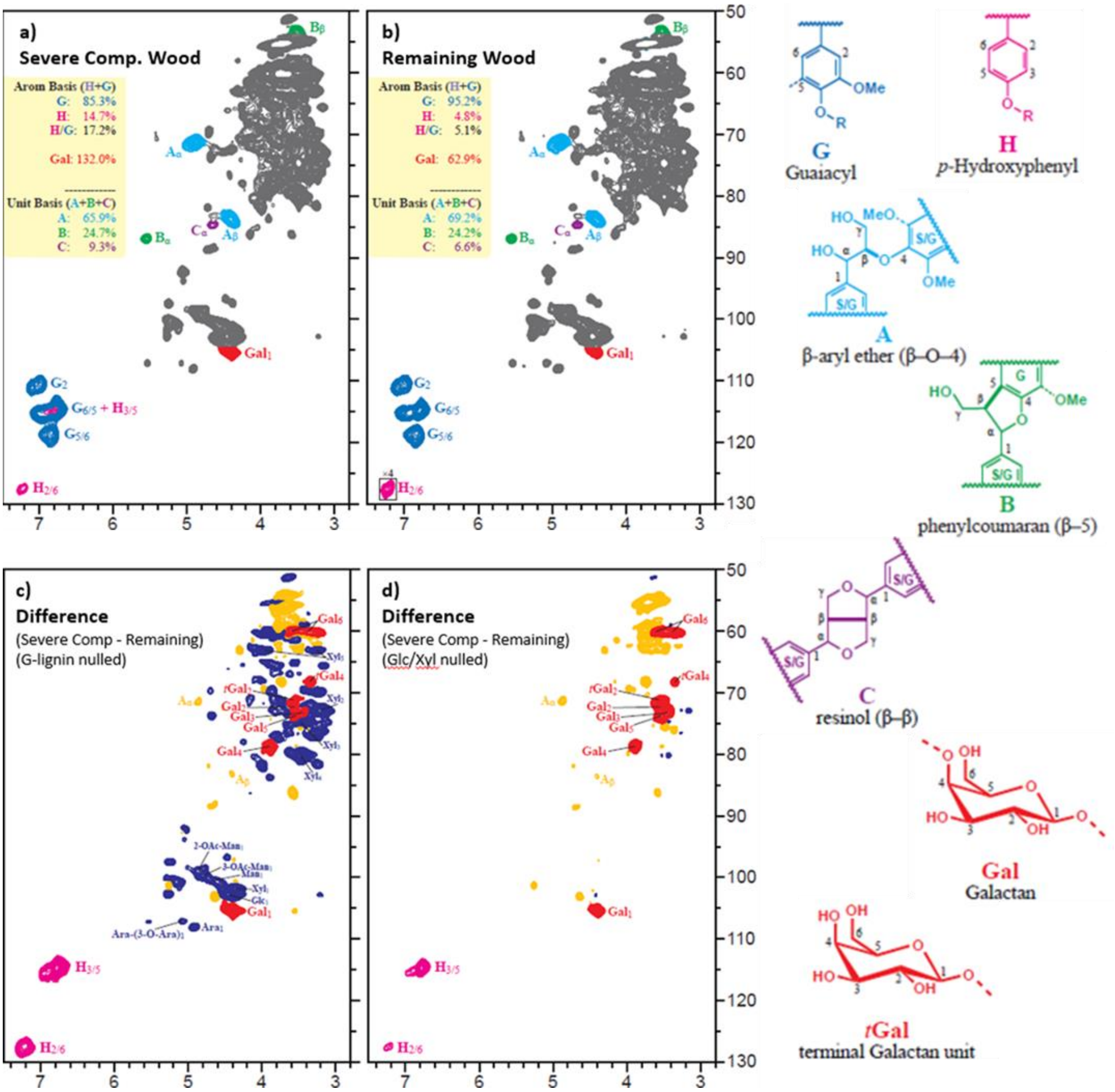

Figure S6: *Comparison HSQC solution-state NMR spectra of* [13]*C compression wood and the remaining wood. Partial whole-cell-wall solution-state HSQC NMR spectra from gels in 4:1 v/v DMSO-d6:pyridine-d5. a) Compression wood, b) Remaining wood, c) Compression wood (1.0) minus remaining wood (1.0) nulling the guaiacyl G2 contour peak showing the substantial H-lignin level increase and concomitant Gal levels increase, d) Compression wood (1.0) minus remaining wood (0.8) nulling the cellulose/xylan C1 contour peaks showing the substantial Gal level increase and concomitant lignin H-levels increase.*

Key peaks are coloured to match the structures shown. 2D difference spectra, with hot colours (red, orange) representing components elevated vs cold colours (dark blue peaks) lowered.

Data in the yellow boxes is from volume-integration of resolved contour peaks: guaiacyl lignin ***G***$_{2}$*, p-hydroxyphenyl lignin* ***H***$_{2/6}$*, β-galactan* ***Gal***$_{1}$*, and the 3 major units resulting from radical coupling during lignification, the β-ether (β–O–4-coupled)* ***A***$_{\alpha}$*, phenylcoumaran (β–5-coupled)* ***B***$_{\alpha}$*, and resinol (β–β-coupled)* ***C***$_{\alpha}$*. Such spectra are not fully quantitative, but differences/ratios are valid. For example, in the compression the galactan (Gal) likely really is 132.0/62.9 = 2.1 times the level on a lignin (H+G) basis in the compression wood vs in the remaining wood.*

H*-levels are over-quantified due to their longer relaxation times as mobile end-units on a less mobile polymer chain. Note that the H-units were not evident at the level plotted so the 4x levels are shown in the box in b) to indicate/validate the presence of (lower levels of)* ***H****-units in the remaining wood.*

Note that the Gal$_{6}$ *proton shifts could not be determined (so the position of that red-coloured peak is not necessarily accurate).*

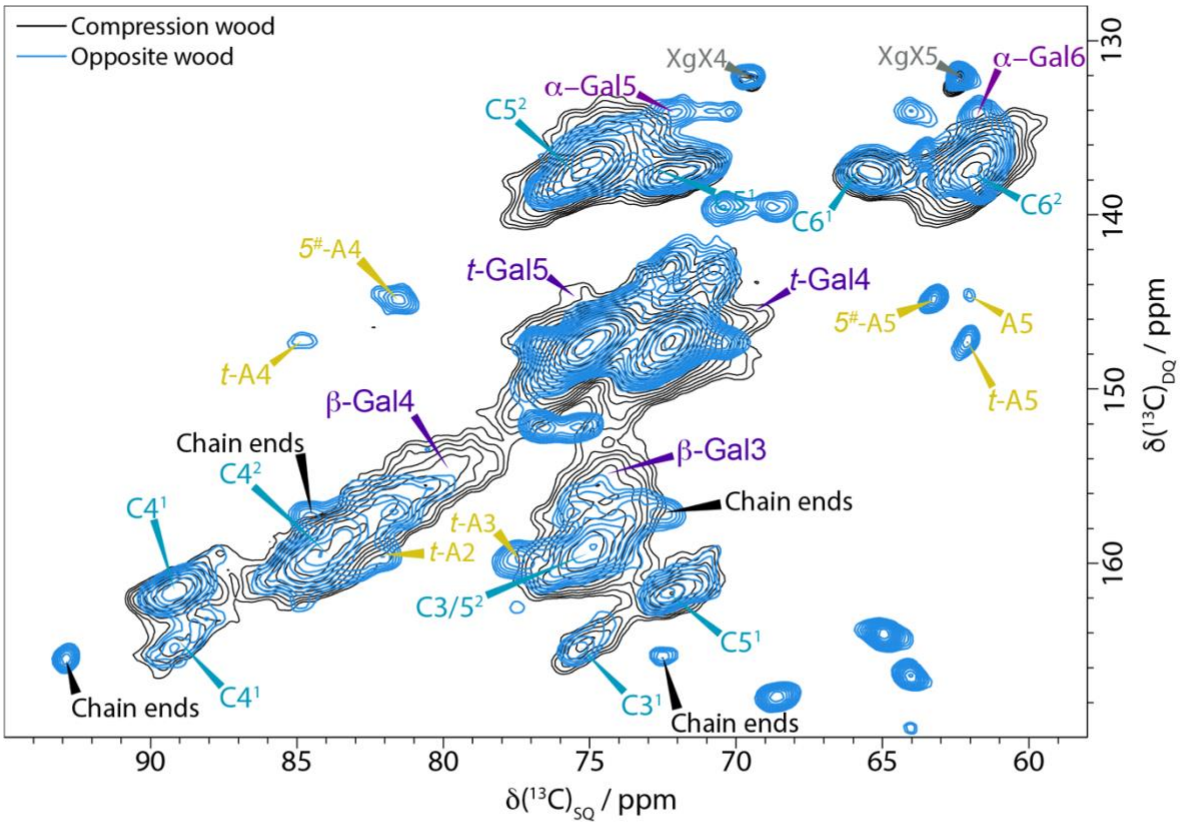

Figure S7: *Comparison of the C4-C5−C6 region of ^{13}C DP-INADEQUATE NMR spectra of compression wood (black) and opposite wood (blue) normalised to the cellulose C4^{1} peak at 89 ppm. The spectra were recorded at a ^{13}C Larmor frequency 150.7 MHz and a MAS frequency of 12 kHz. The spin-echo duration used was 2.2 ms.*

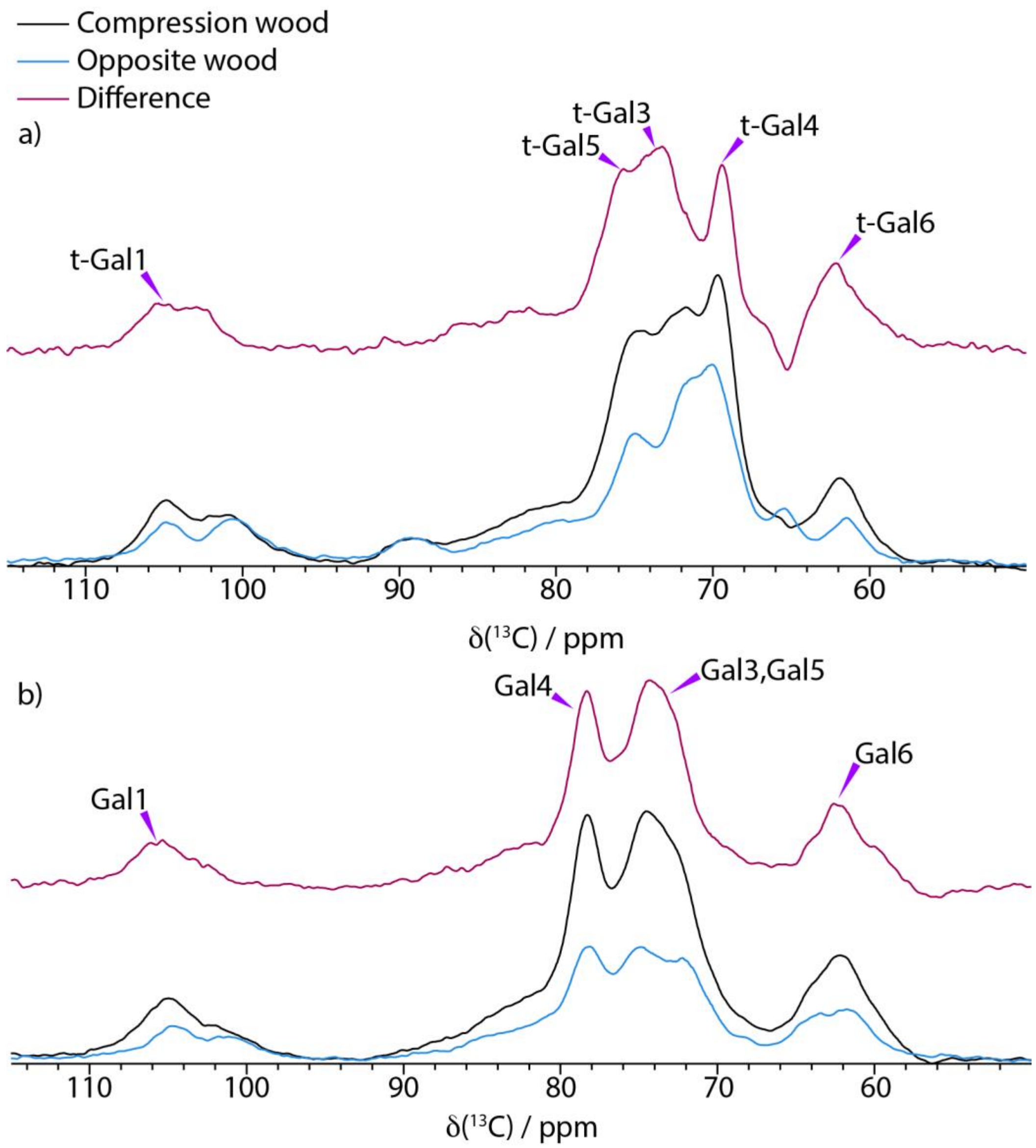

***Figure S8**: Comparison of slices from the 30 ms CP PDSD spectra of compression wood (black) and opposite wood (blue) and the difference (mauve). The spectra were recorded at a ^{13}C Larmor frequency 150.7 MHz and a MAS frequency of 12 kHz. a) The 69.3 ppm slice where the difference reveals the terminal (1→4)-β-galactan shifts. b) The 78.5 ppm slice where the difference reveals the (1→4)-β-galactan shifts.*

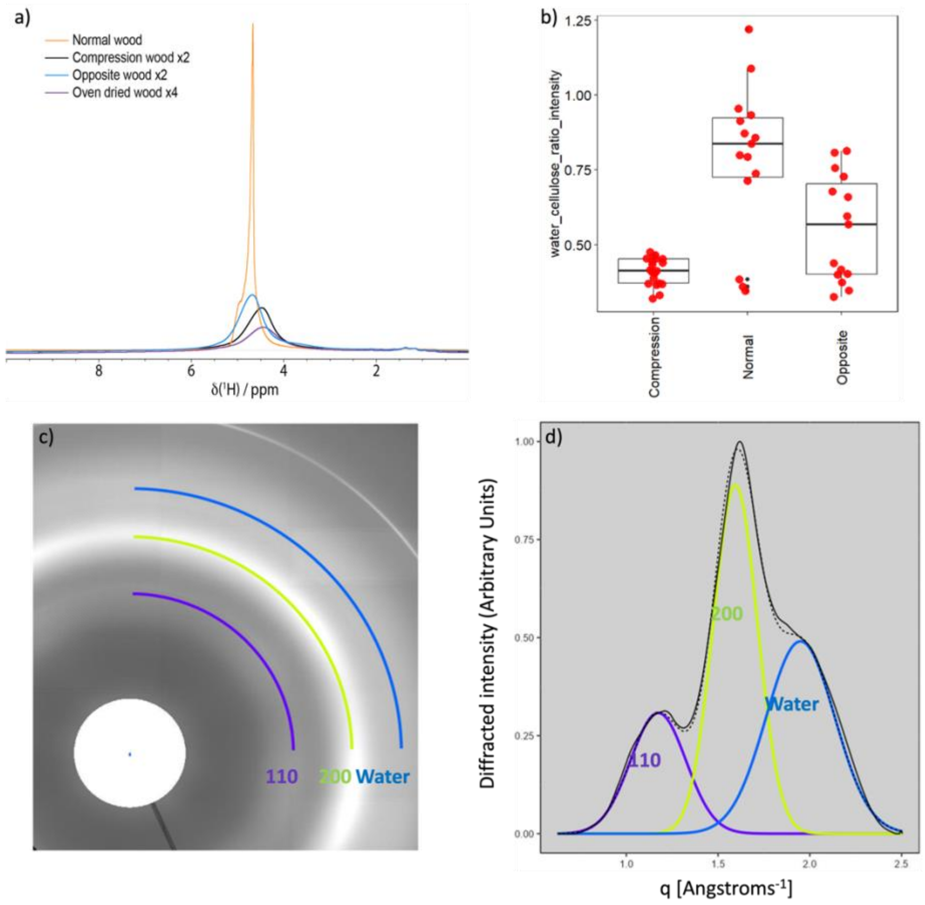

***Figure S9**: a) Comparison of 1D ^{1}H echo spectra of never dried pine (orange), compression wood (black), opposite wood (blue) and oven dried wood (mauve). Spectra were recorded at a ^{1}H Larmor frequency of 600 MHz and a MAS frequency of 12 kHz with an echo time of 1 rotor period. b) Water/cellulose peak intensity ratio measured from X-Ray diffraction experiment. c) Example of X-Ray scattergram of Pinus radiata normal wood. d) Deconvolved peaks based on the intensity of the scattering pattern. The solid black line represents the normalised intensity plot. The coloured lines indicate the deconvolved peaks for the individual components: cellulose 110, cellulose 200, and water. The dashed line represents the summed peaks fit of the modelled deconvolved peaks.*

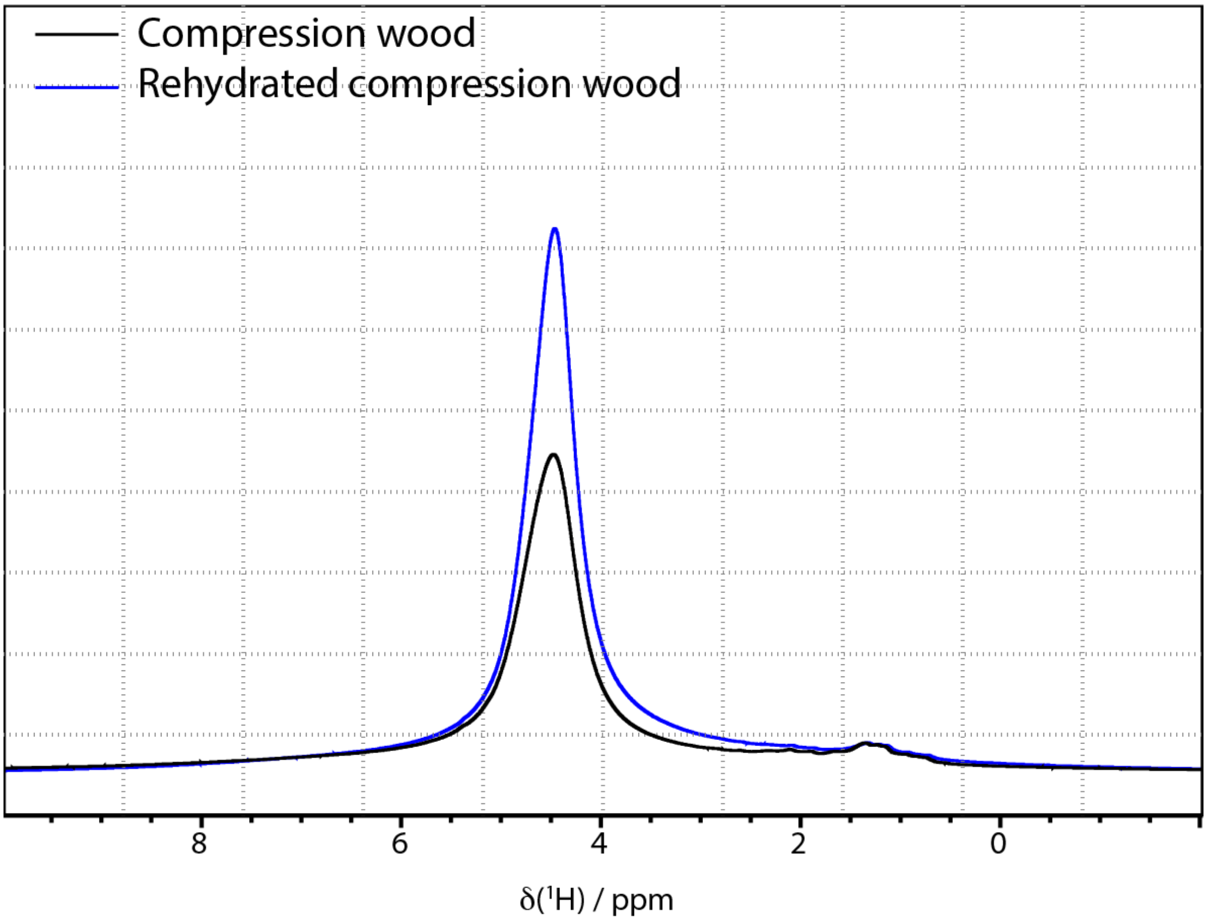

***Figure S10**: Comparison of the 1D ^{1}H spectra of compression wood (black) vs rehydrated compression wood (blue) after 14 days in a 100% humidity atmosphere.*

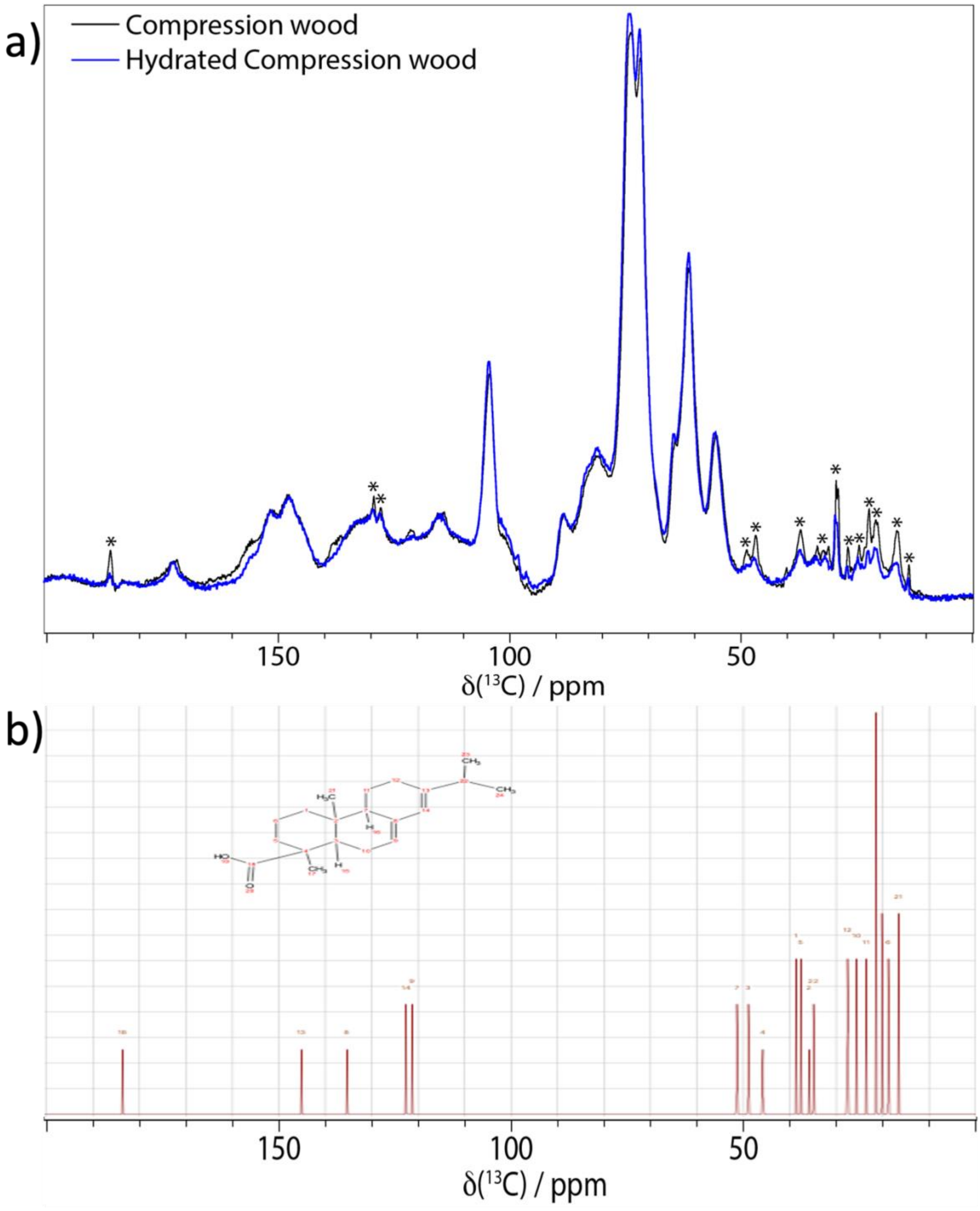

***Figure S11**: a) Comparison of 1D DP 2s spectra of compression wood (black) vs rehydrated compression wood (blue). The ^{13}C shifts of the dehydroabietic acid have been marked with an asterisk. Spectra were recorded at a ^{13}C Larmor frequency of 150.7 MHz and a MAS frequency of 12 kHz. b) in silico prediction of the dehydroabietic acid peak shift.*

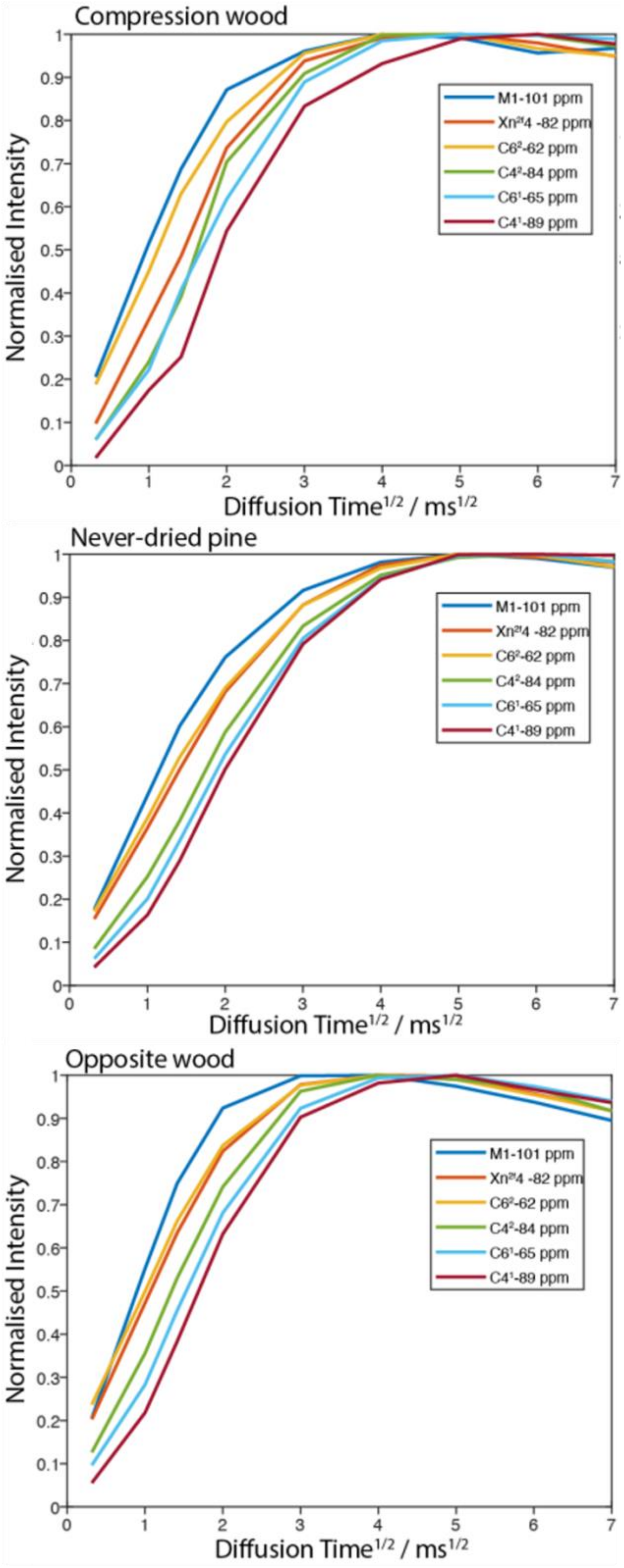

Figure S12*: Comparison of build-up curves of a water-edited experiment for never dried pine, compression wood and opposite wood showing that mannan (blue) is closest to water in all woods. The next closest in compression wood is the galactan and domain 2 cellulose C6 (yellow)*

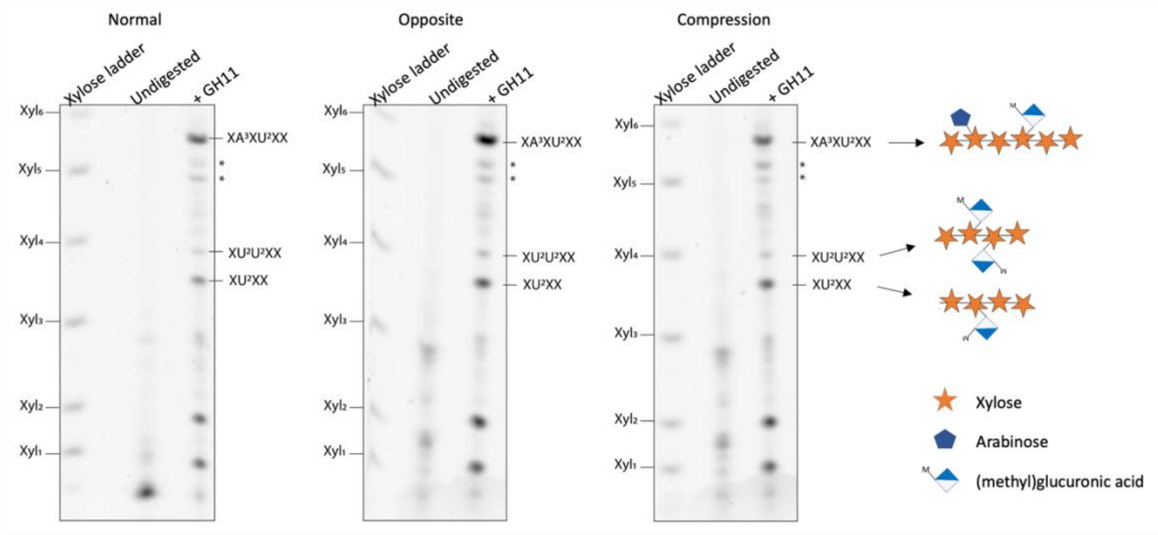

***Figure S13**: Polysaccharide Analysis Using Carbohydrate Gel Electrophoresis (PACE) of hemicelluloses extracted from normal, opposite and compression wood of Pinus radiata AIR cell walls. The extracts are run on the gel either undigested or after a GH11 digestion. The three main oligosaccharides products XA^3XU^2XX, XU^2U^2XX and XU^2XX are represented on the right-hand side of the figure. The asterisks represent unidentified oligosaccharides.*

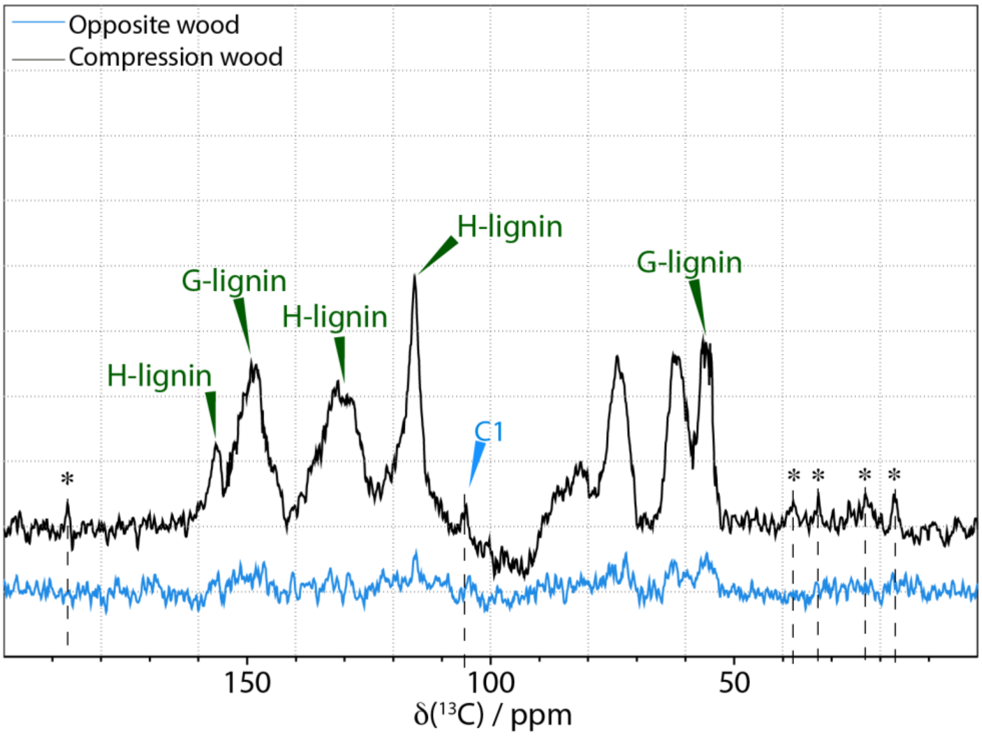

Figure S14: *Comparison of the H lignin peak at 115.5 ppm slice of the 400 ms CP PDSD spectra of compression wood (black) and opposite wood (blue). The visible dehydroabietic acid shifts are indicated with an asterisk. The spectra were recorded at a ^{13}C Larmor frequency 150.7 MHz and a MAS frequency of 12 kHz.*

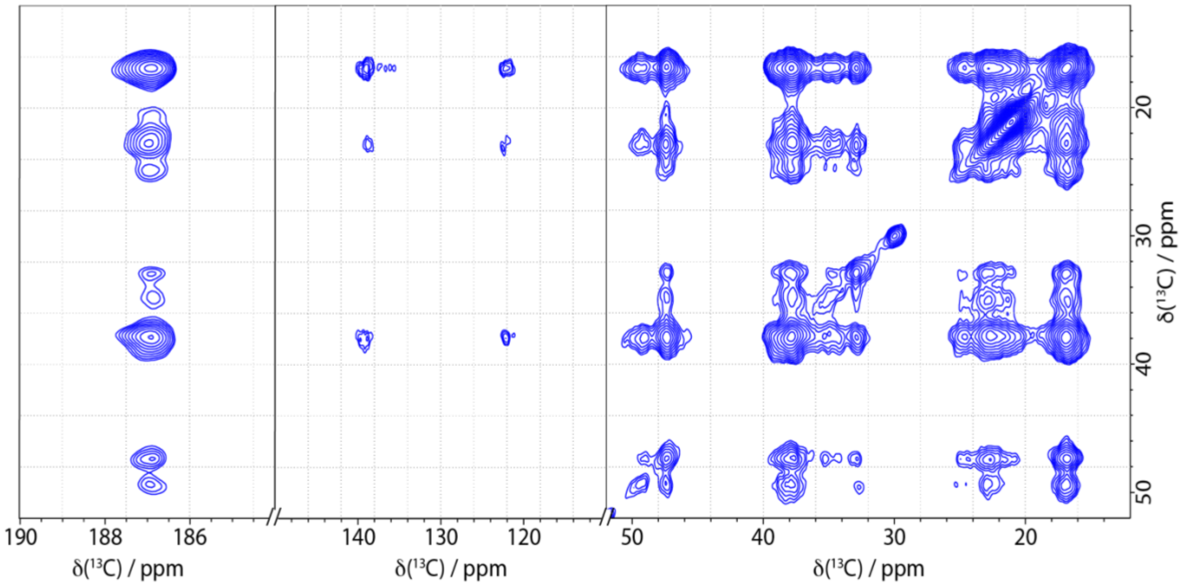

Figure S15: *The aliphatic region of 400 ms ^{13}C CP-PDSD NMR spectrum of compression wood showing the cross peaks for the dehydroabietic acid.*

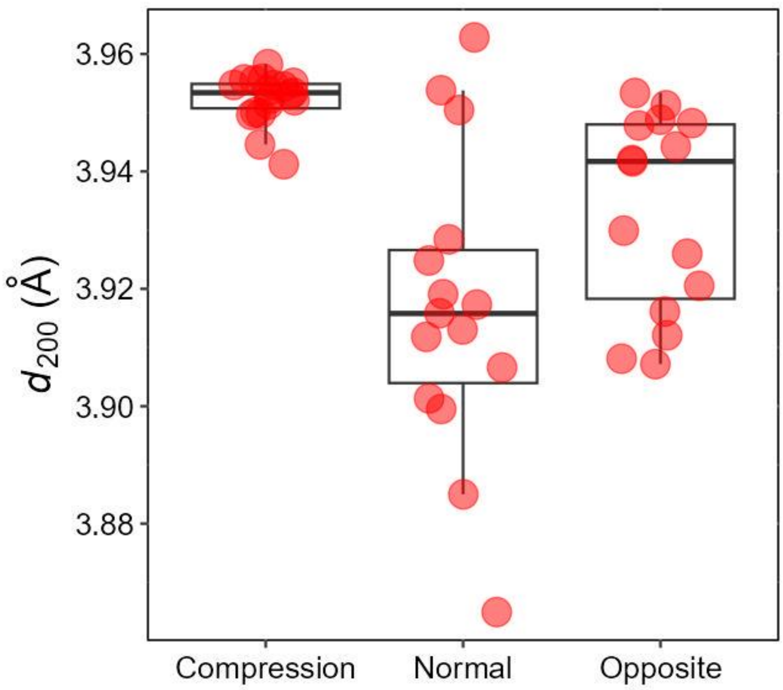

Figure S16: *Calculated d-spacing for the (200) diffraction peak.*

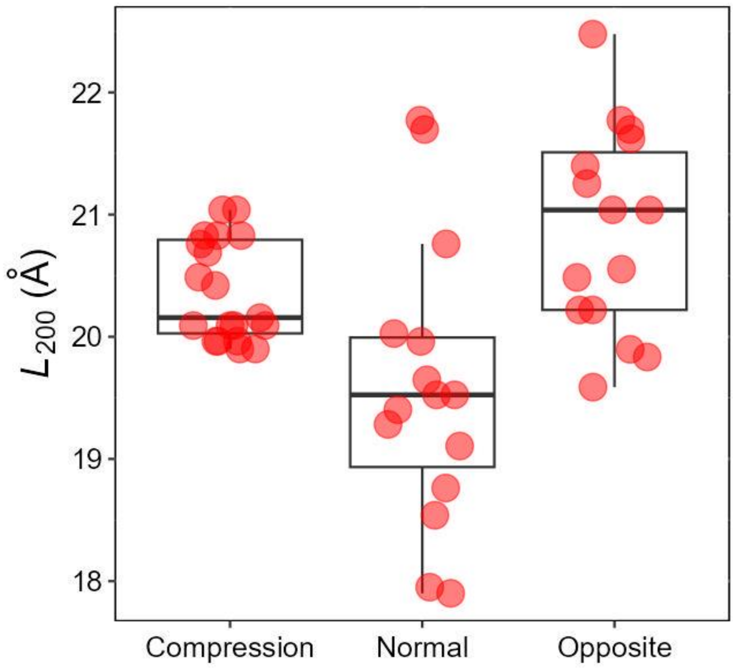

SI Figure S17: *Calculated lateral crystal minimum domain size based on the (200) diffraction.*

Supplementary tables

Table S1: Structural and supramolecular differences found in the normal to severe compression wood continuum.

Wood type	Layers (Underlining indicates presence of lignin)	MFA (°)			S2 Macro-fibril size (nm)	Cellulose crystallinity (%)
		S1	S2	S3		
**Normal / Opposite**	CML / S1 / S2 / S3	70-50	5-30	70-50	19	50
**Mild Compression 1**	CML / S1 / partial S2L / thin S3					
**Mild Compression 2**	ICS / CML / S1 / S2L / S2i					
**Severe compression**	ICS / CML / S1 / S2L (outer S2) / S2i (with helical cavities)	90	30-45	N/A	22	45–50 or 50

N.B. CML: compound mid lamellae, S2L: highly lignified outer S2 layer; S2i: less lignified inner S2 layer; ICS, intercellular space. ((Donaldson and Frankland, 2004, Altaner et al., 2007, Donaldson, 2008, Nanayakkara et al., 2009, Mellerowicz and Gorshkova, 2012)

Table S2: Chemical differences found in the normal to severe compression wood continuum.

Wood	Acetyl (%)	Cellulose (%)	Xylan (%)	Mannan (%)	Total lignin (%)	H (%)	(1→4)-β-galactans (%)	(1→3)-β-glucans (%)
**Normal / Opposite**	1.4	42-51	5-8	13-20	26-28	Trace <1	Trace – 0.74	-
**Mild Compression 1**					28	7	2-3	
**Mild Compression 2**					32	11		
**Severe compression**	0.8	30-38	6-7	8-9	35-40	14	9-10%	3-5%<br>Inner S2i helical cavities

(Timell, 1986, Yeh et al., 2006, Altaner et al., 2007, Harris and Stone, 2009, Altaner et al., 2010, Brennan et al., 2012, Chavan et al., 2015, Zhang et al., 2017, Purusatama et al., 2020)

Step	Start temp	End temp	Isothermal at end temperature duration (min)	Ramp (°C/min)
1	-30	-20	5	1
2	-20	-15	5	1
3	-15	-10	5	1
4	-10	-5	10	1
5	-5	-2	10	1
6	-2	-1	10	1

Table S3: Subsequent ramp/isothermal steps

Table S4: The NMR shifts of galactan identified in compression wood compared with those of domain 2 cellulose. † are known NMR shifts for β -1-4-linked-galacatan and *t*-Galactan which have been assigned by Gao et al., 2023

Saccharide	C1	C2	C3	C4	C5	C6
Compression wood galactan	105.3	-	-	78.5	-	62.0
Compression wood *t*-Galactan	104.7	-	73.8	69.5	76.0	62.2
Cellulose domain 2	105.1	72.0	-	83.5	75.2	61.7
	105.3	72.0	-	84.6	75.0	62.2
β -1-4-linked-galactan†	105.1	73.0	74.3	78.5	75.5	61.9
t-Galactose†	104.3	71.9	73.9	69.5	75.8	62.0

Table S5:^{13}C solid-state NMR T_1 relaxation times for all three wood samples using a fit to a single exponential. An asterisk indicates those where this fit was not good. Error in the T_1 relaxation times is ±10 %

Integrated peak area	Normal wood T_1 (s)	Compression wood T_1 (s)	Opposite wood T_1 (s)
**C1 – 105 ppm**	4.7	5.4*	5.6
**M1 – 101 ppm**	1.8	3.9	2.9*
**$C4^1$ – 89 ppm**	5.91	10.2*	7.3*
**$C4^2$ – 84 ppm**	3.8	5.7	4.2
**2-fold xylan – 82 ppm**	3.1	4.8	3.5
**$C6^1$ – 65 ppm**	4.1	5.5	4.4
**$C6^2$ -62 ppm**	2.7	3.6	2.8*
**Lignin methoxy – 56 ppm**	3.2	3.6	3.8
**Lignin aromatic – 150 ppm**	3.8	5.2	6.7*

Table S6: Mean value of the water/cellulose peak intensity ratio from XRD together with the statistical analysis. The Kruskel-Wallis test, a non-parametric test analogous to ANOVA, compares the three group simultaneously. The bold numbers represent significant difference between the types of wood. The Wilcoxon test compares the wood types by pair.

	Mean
Compression	0.411
Normal	0.787
Opposite	0.553

Kruskel-Wallis	Compression	Normal	Opposite
Compression	2.000	1.000	1.139
Normal	**0.000**	2.000	1.034
Opposite	0.139	**0.034**	2.000

Wilcoxon	P Value
Compression < Normal	0.000
Compression = Opposite	0.063
Opposite < Normal	0.008

Table S7: Comparison of hydration of all wood samples and ^{1}H NMR T_2 relaxation times for all wood samples fit to a single exponential.

Sample	Integral of water peak compared to normal wood	^{1}H T_2 of water / ms
**Normal pine**	100%	6.9
**Compression wood**	42%	0.62
**Opposite wood**	43%	0.70
**Oven dried wood**	21%	0.45

Table S8: Mean d_{200} value with statistical analysis. The Kruskel-Wallis test, a non-parametric test analogous to ANOVA, compares the three group simultaneously. The bold numbers represent significant difference between the types of wood. The Wilcoxon test compares the wood types by pair.

	**Mean (Å)**
Compression	3.952
Normal	3.917
Opposite	3.933

**Kruskal-Wallis**	**Compression**	**Normal**	**Opposite**
Compression	2.000	1.000	1.000
Normal	**0.000**	2.000	1.347
Opposite	**0.000**	0.348	2.000

**Wilcoxon**	**P Value**
Compression ≠ Normal	**0.004**
Compression ≠ Opposite	**0.000**
Normal = Opposite	0.081

Table S9: Mean minimal lateral crystal domain size L_{200} with associated statistical analysis. The Kruskal-Wallis test, a non-parametric test analogous to ANOVA, compares the three group simultaneously. The bold numbers represent significant difference between the types of wood. The Wilcoxon test compares the wood types by pair.

	**Mean (Å)**
Compression	20.37
Normal	19.59
Opposite	20.87

**Kruskal-Wallis**	**Compression**	**Normal**	**Opposite**
Compression	2.000	1.014	1.208
Normal	**0.014**	2.000	1.000
Opposite	0.208	**0.000**	2.000

**Wilcoxon**	**P Value**
Compression ≠ Normal	**0.004**
Compression = Opposite	0.092
Opposite ≠ Normal	**0.002**

References Supplemental Information

ALTANER, C., HAPCA, A. I., KNOX, J. P. & JARVIS, M. C. 2007. Detection of β-1-4-galactan in compression wood of Sitka spruce [*Picea sitchensis* (Bong.) Carrière] by immunofluorescence. *Holzforschung,* Vol. 61, pp. 311–316.

ALTANER, C. M., TOKAREVA, E. N., JARVIS, M. C. & HARRIS, P. J. 2010. Distribution of (1→ 4)-β-galactans, arabinogalactan proteins, xylans and (1→ 3)-β-glucans in tracheid cell walls of softwoods. *Tree Physiology,* 30, 782-793.

BRENNAN, M., MCLEAN, J. P., ALTANER, C. M., RALPH, J. & HARRIS, P. J. 2012. Cellulose microfibril angles and cell-wall polymers in different wood types of *Pinus radiata*. *Cellulose,* 19, 1385-1404.

CHAVAN, R. R., FAHEY, L. M. & HARRIS, P. J. 2015. Quantification of (1→ 4)-β-D-galactans in compression wood using an immuno-dot assay. *Plants,* 4, 29-43.

DONALDSON, L. 2008. Microfibril angle: measurement, variation and relationships – a review. *Iawa Journal,* 29, 345-386.

DONALDSON, L. & FRANKLAND, A. 2004. Ultrastructure of iodine treated wood. *Holzforschung,* 58, 219-225.

HARRIS, P. J. & STONE, B. A. 2009. Evolutionary aspects of (1, 3)-β-glucans and related polysaccharides. *Chemistry, Biochemistry, and Biology of 1-3 Beta Glucans and Related Polysaccharides.* Elsevier.

MELLEROWICZ, E. J. & GORSHKOVA, T. A. 2012. Tensional stress generation in gelatinous fibres: a review and possible mechanism based on cell-wall structure and composition. *Journal of experimental botany,* 63, 551-565.

NANAYAKKARA, B., MANLEY-HARRIS, M., SUCKLING, I. D. & DONALDSON, L. A. 2009. Quantitative chemical indicators to assess the gradation of compression wood. *Holzforschung,* 63, 431-439.

PURUSATAMA, B. D., CHOI, J. K., LEE, S. H. & KIM, N. H. 2020. Microfibril angle, crystalline characteristics, and chemical compounds of reaction wood in stem wood of Pinus densiflora. *Wood Science and Technology,* 54, 123-137.

TIMELL, T. E. 1986. *Compression wood in gymnosperms.* Springer.

YEH, T.-F., BRAUN, J. L., GOLDFARB, B., CHANG, H.-M. & KADLA, J. F. 2006. Morphological and chemical variations between juvenile wood, mature wood, and compression wood of loblolly pine (*Pinus taeda* L.). *Holzforschung,* 60, 1-8.

ZHANG, M., LAPIERRE, C., NOUXMAN, N. L., NIEUWOUDT, M. K., SMITH, B. G., CHAVAN, R. R., MCARDLE, B. H. & HARRIS, P. J. 2017. Location and characterization of lignin in tracheid cell walls of radiata pine (*Pinus radiata* D. Don) compression woods. *Plant Physiology and Biochemistry,* 118, 187-198.
